# T cell receptor recognition of hybrid insulin peptides bound to HLA-DQ8

**DOI:** 10.1038/s41467-021-25404-x

**Published:** 2021-08-25

**Authors:** Mai T. Tran, Pouya Faridi, Jia Jia Lim, Yi Tian Ting, Goodluck Onwukwe, Pushpak Bhattacharjee, Claerwen M. Jones, Eleonora Tresoldi, Fergus J. Cameron, Nicole L. La Gruta, Anthony W. Purcell, Stuart I. Mannering, Jamie Rossjohn, Hugh H. Reid

**Affiliations:** 1 Infection and Immunity Program & Department of Biochemistry and Molecular Biology, Biomedicine Discovery Institute, Monash University, Clayton, VIC Australia; 2 Australian Research Council Centre of Excellence in Advanced Molecular Imaging, Monash University, Clayton, VIC Australia; 3 Immunology and Diabetes Unit, St Vincent’s Institute of Medical Research, Fitzroy, VIC Australia; 4 Department of Endocrinology and Diabetes, Royal Children’s Hospital, Murdoch Children’s Research Institute, Parkville, VIC Australia; 5 Department of Paediatrics, University of Melbourne, Melbourne, VIC Australia; 6 Institute of Infection and Immunity, Cardiff University, School of Medicine, Heath Park, Cardiff, UK

**Keywords:** Autoimmunity, X-ray crystallography

## Abstract

HLA-DQ8, a genetic risk factor in type I diabetes (T1D), presents hybrid insulin peptides (HIPs) to autoreactive CD4+ T cells. The abundance of spliced peptides binding to HLA-DQ8 and how they are subsequently recognised by the autoreactive T cell repertoire is unknown. Here we report, the HIP (*GQVELGGG*NAVEVLK), derived from splicing of insulin and islet amyloid polypeptides, generates a preferred peptide-binding motif for HLA-DQ8. HLA-DQ8-HIP tetramer^+^ T cells from the peripheral blood of a T1D patient are characterised by repeated *TRBV5* usage, which matches the TCR bias of CD4+ T cells reactive to the HIP peptide isolated from the pancreatic islets of a patient with T1D. The crystal structure of three TRBV5+ TCR-HLA-DQ8-HIP complexes shows that the *TRBV5*-encoded TCR β-chain forms a common landing pad on the HLA-DQ8 molecule. The N- and C-termini of the HIP is recognised predominantly by the TCR α-chain and TCR β-chain, respectively, in all three TCR ternary complexes. Accordingly, TRBV5 + TCR recognition of HIP peptides might occur via a ‘polarised’ mechanism, whereby each chain within the αβTCR heterodimer recognises distinct origins of the spliced peptide presented by HLA-DQ8.

## Abstract (web summary)

Epitopes formed by fusion of more than one self peptide, such as proinsulin and other β cell proteins, can result in the formation of non-self hybrid peptides that can potentially trigger autoimmune responses. Here the authors show how TRBV5 + T cell receptors are geared towards recognition of HLA-DQ8 bound hybrid peptides in patients with type 1 diabetes.

## Introduction

The haplotypes encoding HLA-DR3/DQ2 (DRB1*03:01-DQA1*05:01-DQB1*02:01) and HLA-DR4/DQ8 (DRB1*04:01-DQA1*03:01-DQB1*03:02) allomorphs confer very high risk of developing type I diabetes (T1D), with an odds ratio of >3.6 and 11.0, respectively^1^. Within these haplotypes the HLA-DQ alleles, HLA-DQ2 and -DQ8, confer the majority of the risk^2^. Human islet-infiltrating CD4+ T cells recognise several epitopes derived from the proinsulin C-peptide presented by HLA-DQ8^3,4^. Such studies provide some evidence to suggest that CD4+ T cell responses against proinsulin play a role in human T1D, although the extent of the autoreactive epitopes that can precipitate the disease remain unknown.

It has been reported that murine CD4+ T cells and human islet-infiltrating CD4+ T cells recognise β-cell derived epitopes formed by the fusion of proinsulin C-peptide with other β-cell granule proteins^5–10^. This type of post-translational modification (PTM) dramatically changes the peptide sequence by joining two ‘self’ peptides together to form a ‘non-self’ hybrid peptide. Peptide splicing is a novel pathway for generating CD4 + and CD8 + T cell epitopes^5–13^. Protease-mediated peptide splicing, or transpeptidation, has been described in vitro^14^, in bacteria^15^, in plants^16^, and in humans^5^. For example, in humans, proteasome-mediated protein splicing generates epitopes recognised by tumour specific CD8 + T cells^11–13^. In the context of T1D, such spliced epitopes are referred to as ‘hybrid insulin peptides’ (HIPs), representing fusions of (pro)insulin with other β-cell proteins. HIP formation in beta cells may be favoured because β-cell granules are densely packed with insulin, C-peptide and several other proteins including chromogranins and amyloid proteins^17^. Furthermore, the β-cell granules are the site of active proteolysis, where in addition to proinsulin, many proteins, including chromogranins and IAPP, are cleaved by the granule proteases to generate an array of bio-active peptides^17^. Cleaved C-peptide is further digested to give numerous proinsulin C-peptide fragments that can be detected by mass spectrometry^5,10^. Interestingly, a mechanism for hybrid peptide formation in T1D has been described by Reed et al.^18^. Here, neo-antigens for diabetogenic CD4 T cells were generated by transpeptidation via lysosomal protease cathepsin L reverse proteolysis of secretory granule protein fragments^18^.

A study of human islet-infiltrating CD4+ T cell clones isolated from the residual pancreatic islets of a deceased organ donor who had T1D, showed responses to proinsulin C-peptide^3,4^. In addition to C-peptide, HIPs presented by HLA-DQ8 also stimulated responses from some of these human islet-infiltrating CD4+ T cell clones^10^. The HIPs described were formed by the fusion of proinsulin C-peptide fragments (GQVELGGG) with the β-cell granule protein IAAP2 peptide fragment (NAVEVLK). While some of the pancreatic islet-infiltrating CD4+ T cells responded to the proinsulin C-peptide, the C-peptide-IAPP2 HIP presented by HLA-DQ8 (*GQVELGGG*NAVEVLK) may potentially be a more effective antigen due to the generation of a more favourable HLA-DQ8 binding motif. Specifically, this HIP contains a glutamic acid residue (E) at peptide positions 1 and 9 (underlined), which are the favoured ‘anchor’ residues for HLA-DQ8 restricted peptide antigens^19,20^. The discovery of HIPs as target neoantigens for the immune response in T1D provides a possible explanation of how T cells, that escape negative-selection in the thymus, can manifest autoreactivity towards β-cell antigens.

There is little understanding of the molecular basis underpinning T cell receptor (TCR) recognition of HLA-DQ8-HIP complexes. Here we demonstrate the natural repertoire of HLA-DQ8 hybrid peptides within an antigen presenting cell (APC). We examine the T cell repertoire in PBMCs from T1D patients stimulated by HIP peptides and observed a *TRBV5* + biased immune response to HLA-DQ8-HIP. We present three structures of the *TRBV5* + TCRs in complex with HLA-DQ8-HIP, revealing a common pattern of recognition, whereby the TCR α-chain and β-chain interacted with the N- and C-terminal regions of the HIP peptide, respectively, thereby suggesting a ‘polarised’ mechanism of HIP recognition by TRBV5 + TCRs.

## Results

### High abundance of spliced peptides presented by HLA-DQ8

**Fig. 1:**
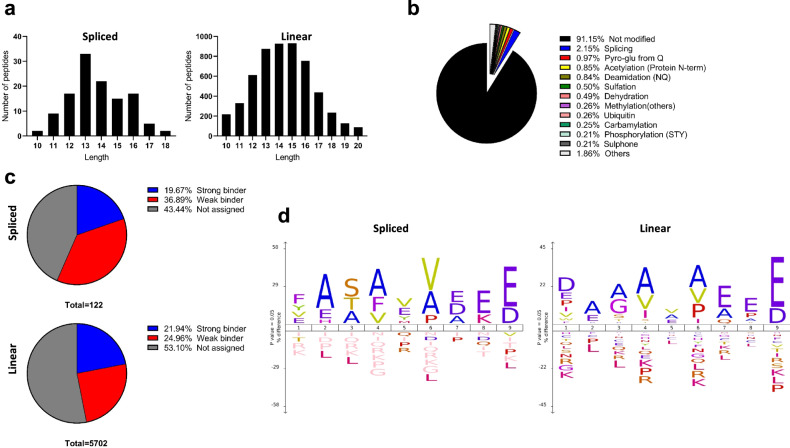
Spliced peptides in the HLA-DQ8 peptide repertoire. Peptide ligands identified from immunoaffinity purified HLA-DQ8 isolated from 9033 cells showing: **a** spliced and linear peptide length distributions; **b** Proportion and types of PTMs observed in peptidome; **c** Estimated HLA-DQ-8 affinity of spliced and linear peptides; **d** spliced and linear peptide core-binding 9-mer motifs.

We first examined the relative abundance of spliced peptides being presented by HLA-DQ8 in comparison to non-spliced peptides. HLA-DQ8 molecules were affinity purified with anti-HLA-DQ immunoaffinity chromatography from the 9033 EBV transformed B lymphoblastoid cell line and HLA-DQ8 bound peptides were pre-fractionated by HPLC prior to mass spectrometry analysis^21^. Data was analysed by Peaks X-plus software and hybrid finder algorithm. We identified a total of 6206 peptides, derived from 1079 source proteins, presented by HLA-DQ-8 by the 9033 BLCL cells at a false discovery rate (FDR) of 1% (Supplementary Data 1). As expected for HLA class II epitopes, the majority of peptides (5441 peptides; ~88.7%) were between 10–18 amino acids in length with no apparent difference between linear and spliced sequences (Fig. 1a). From these 5441 peptides, approximately 400 peptides were assigned as chemically modified (we removed oxidation as this modification could be an artefact arising from the sample preparation process) and 122 peptides were assigned as spliced peptides. Spliced peptides represented ~20% of the total PTM peptides (spliced and chemically modified) and ~2% of all identified peptides (10–18 amino acids in length) (Fig. 1b). The 2% spliced peptides were based on unique sequences (i.e., a frequency based estimate not an abundance based estimate) and thus were not dominated by a favoured highly abundant peptide (Supplementary Data 1). Using NetMHCIIpan 4.0 as a prediction tool, a similar proportion of binding peptides (score < 2: strong binder; <10: weak binder; ≥10 not assigned as HLA-DQ-8 binder) was observed for both linear (~47%) and spliced (~56%) peptides (Fig. 1c). We also found a similar motif for the 9-mer core sequence for both linear and spliced peptides (Fig. 1d). Hence, in an antigen-presenting cell line cultured in vitro, peptide splicing forms hybrid peptides with the preferred HLA-DQ8 anchor residues. We have demonstrated that ~2% MHC-II bound peptide species are assigned as hybrid peptides produced constitutively in BLCLs.

### Comparison of proinsulin C-peptide and HIP reactivity by islet-infiltrating T cells

**Fig. 2:**
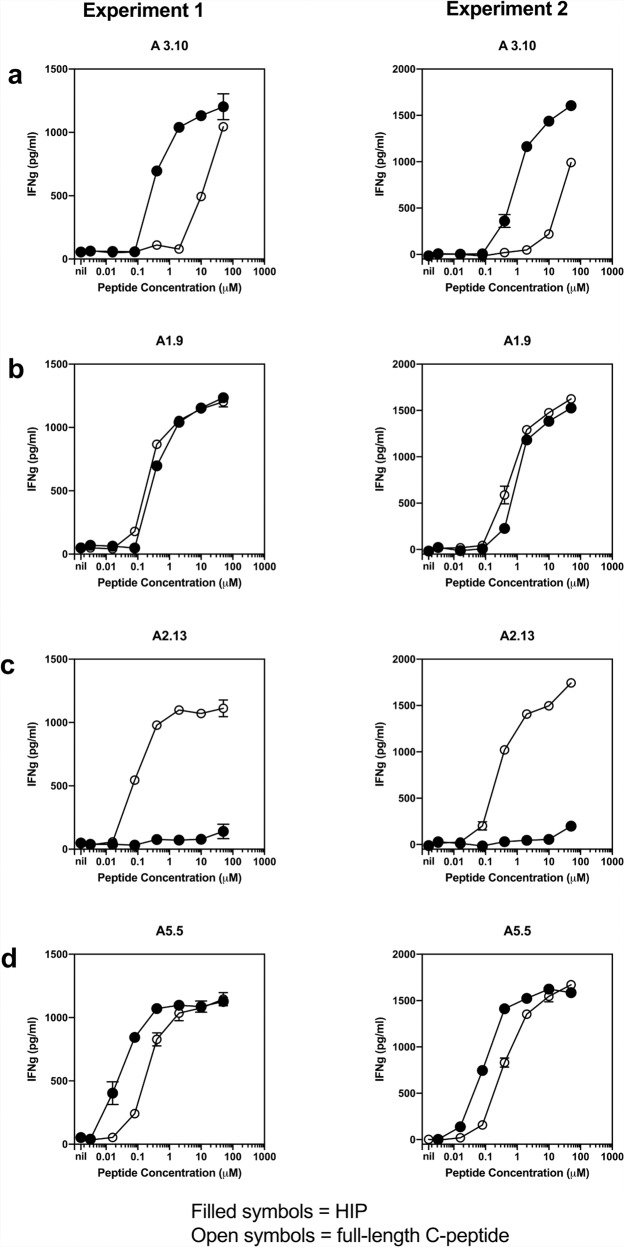
Comparison of the stimulatory capacity of full-length proinsulin C-peptide and HIP on T1D CD4 + T cell clones. A different clone is shown in each panel, clone A3.10 (**a**), clone A1.9 (**b**), clone A2.13 (**c**), clone A5.5 (**d**). Titration of full-length 31-mer C-peptide (EAEDLQVGQVELGGGPGAGSLQPLALEGSLQ, (open circles) and 15mer C-peptide-IAPP2 HIP (GQVELGGGNAVEVLK (filled circles). Antigen presenting cells (APC) were the EBV line, EBV-KJ, (2 × 104 cells/well). APC were cultured for 24 h with the same number (2 × 10^4^ cells/well) of the primary CD4 + T-cell clone and the peptide concentration indicated. T-cell response to peptide were indicated by secretion of IFNγ which was measured in triplicate by ELISA and reported as pg/ml. Mean and standard deviations of triplicate IFNγ measurements are shown. Results are representative of two experiments for each clone.

Human islet-infiltrating CD4+ T cell clones were previously isolated from the residual pancreatic islets of a deceased T1D organ donor, all of which recognised the same proinsulin C-peptide core region (GQVELGGGPGAG) presented by HLA-DQ8^4^. With the discovery of the C-peptide-IAPP2 HIP, given the similarity between the HIP sequence and the proinsulin C-peptide, one of these T cell clones, A3.10, was subsequently tested in a T cell stimulation assay and shown to be 100 times more sensitive to C-peptide-IAPP2 HIP than the proinsulin C-peptide^10^. The gene usage of clone A3.10 is TRAV38-1*03-TRBV5-1*01 (previously described in ref. ^4^ and Supplementary Table 1). Four other proinsulin C-peptide reactive T cell clones from the original study shared the same TRBV5-1*01 gene usage. Specifically, three clones (A2.13, A5.5, and A5.8) used TRAV26-1*01-TRBV5-1*01, while the fourth clone A1.9 used TRAV20*02-TRBV5-1*01 (previously described in ref. ^4^ and Supplementary Table 1). Given their shared *TRBV5-1* gene usage with A3.10, we investigated the epitope specificity of the remaining four CD4+ T cells for HLA-DQ8 presenting native proinsulin C-peptide and HIP. The sensitivity of clone A3.10 to the HIP (GQVELGGGNAVEVLK) compared to native proinsulin C-peptide was repeated (Fig. 2a) for direct comparison to the other T cell clones used here (Fig. 2b–d). The other clones have differing sensitivities to the HIP. Clone A1.9 responds equally to proinsulin C-peptide or HIP (Fig. 2b), whereas clone A2.13 very weakly responded to the HIP peptide (Fig. 2c). Clone A5.5 was ten times more sensitive to the HIP peptide than native proinsulin C-peptide (Fig. 2d). Given the inherent technical challenges of working with primary T cell clones, to gain a better understanding of the TCR specificity of these cells, we generated a panel of SKW-3 T cell lines transduced with the TCRs of the original primary clones. TCR reactivity for a given HLA-DQ8-peptide was measured by upregulation of the early T cell activation marker CD69. This reactivity in a transduced T cell line is a measure of the peptide-HLA restriction of the TCR as well as its ability to transduce an activation signal in isolation, as opposed to using primary T cell clones where the presence or absence of inhibiting or activating co-receptors, respectively, may occur. Hence, the reactivity observed does not signify the pathogenicity of any given TCR’s parental T cell clone. The SKW-3 cells transduced with the clone A3.10 TCR and clone A5.5 TCR showed the same preference for HIP over C-peptide as their respective parental T cell clones (Fig. 2, Supplementary Fig. 1). However, the SKW-A3.10 TCR cells were ~6000 fold more sensitive to the HIP over C-peptide as opposed to 10 fold for the parental clone. Likewise, the SKW-3 A5.5 cells were ~3000 fold more sensitive to the HIP over C-peptide. The A1.9 and A2.13 TCRs differed significantly in peptide reactivity from their parental clones too. SKW-3 A1.9 TCR cells had a 25 fold preference for HIP over C-peptide whilst the parental clone showed no preference. SKW-3 A2.13 TCR cells were five fold more sensitive to HIP than C-peptide whereas the parental clone only very weakly recognised the HIP. Accordingly, the TCR specificity of human islet-infiltrating CD4+ T cell clones show a broad degree of cross reactivity between the HIP and the unmodified proinsulin C-peptide.

### TCR affinity towards HLA-DQ8-HIP

**Fig. 3:**
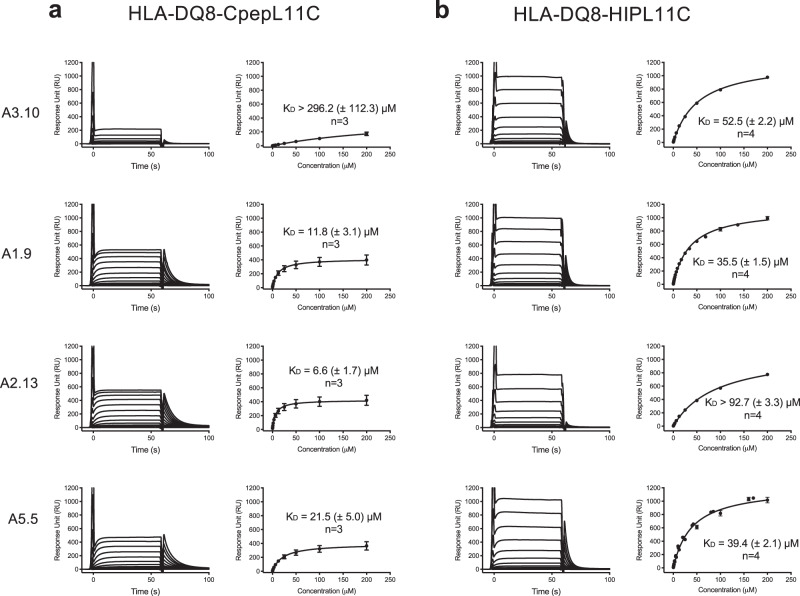
Affinity determination for the HLA-DQ8-CpepL11C/HIPL11C restricted TCRs. Binding analysis of TCRs A3.10, A1.9, A2.13, and A5.5 for **a** HLA-DQ8-CpepL11C and **b** HLA-DQ8-HIPL11C was determined using SPR. A concentration series from 200 μM to 0 μM of each TCR was passed over surface immobilised **a** HLA-DQ8-CpepL11C and **b** HLA-DQ8-HIPL11C. Left columns in **a** and **b**: measured response curves of the single dilution series for each TCR. Right columns in **a** and **b**: Curve fits for TCR-HLA-DQ8-CpepL11C/HIPL11C and K_D_ determination using single ligand binding model. Each TCR sample was analysed in duplicate, and three (*n* = 3) or four (*n* = 4) (as indicated) independent experiments were conducted and measurements were combined after normalising each equilibrium response curve against the calculated response maximum. Data are mean ± standard error, s.e.m. Source data are provided as a Source Data file.

The proinsulin C-peptide epitope (PI_40-54_ GQV**ELGGGPGAG**SLQ; predicted binding register in bold) lacks both a preferred acidic residue at P9 and aliphatic residue at P4, whereas HIP (GQVELGGGNAVEVLK) lacks the preferred P4 residue. Residues at these non-preferred anchor positions led to unstable HLA-DQ8 binary complexes, as judged by size exclusion chromatography (SEC) where a high ratio of aggregate versus the monomeric form of HLA-DQ8 presenting these peptides was observed (not shown). To overcome this, as undertaken previously with a modified B:11-23 peptide and HLA-DQ8^22^, we engineered a construct with a disulfide bond between p11 of the HIP peptide or proinsulin C-peptide and HLA-DQ8α I72C to produce HLA-DQ8αΙ72C-CpepL11C (HLA-DQ8-CpepL11C; GQV**ELGGGPGAG**S*C*Q; predicted binding register in bold, P11Cys in italics) and HLA-DQ8αΙ72C-HIPL11C (HLA-DQ8-HIPL11C; GQV**ELGGG*NAVE***V*C*K; predicted binding register in bold, proinsulin C-peptide derived underlined, IAPP2 derived bold italics and P11Cys italics). These constructs had much lower ratios of aggregate to monomer as judged by SEC (not shown), and Circular Dichroism^2^ showed that the HLA-DQ8-HIP and HLA-DQ8-HIPL11C had the same spectrums, demonstrating that the P11 cysteine substitution did not alter the conformation of the protein (Supplementary Fig. 2). Next, we expressed and purified the five TCRs (A3.10, A1.9, A2.13, A5.5, and A5.8) and used surface plasmon resonance^23^ to determine the binding affinity and level of cross-reactivity of these TCRs towards HLA-DQ8-HIP, -HIPL11C and -CpepL11C. The affinity values of the TCR A3.10, A1.9, and A5.5 for both HLA-DQ8-HIP and the HLA-DQ8-HIPL11C mutant were similar, which is indicative that the HIP-L11C mutation had no influence on TCR recognition (Supplementary Table 2, Supplementary Fig. 3 and Fig. 3).

All five TCRs A3.10, A1.9, A2.13, A5.5 and A5.8 responded to HLA-DQ8-CpepL11C (Fig. 3a, Supplementary Fig. 4a and Supplementary Table 2). Moreover, four out of five of these TCRs (A3.10, A1.9, A2.13, and A5.5) were, to varying extent, cross-reactive, to both HLA-DQ8-CpepL11C and HLA-DQ8-HIPL11C (Fig. 3a, b and Supplementary Table 2). The steady-state affinity (*K*_D_) of TCR A3.10 for HLA-DQ8-HIPL11C was higher with a *K*_D_ of 52.5 ± 2.2 μM compared to *K*_D_ > 296.2 ± 112.3 μM for HLA-DQ8-CpepL11C. The A5.8 TCR only interacted with HLA-DQ8-CpepL11C (*K*_D_ 17.3 ± 4.4 μM) (Fig. 3a, b, Supplementary Fig. 4, and Supplementary Table 2). Like the A5.5 TCR, the A1.9 TCR had similar affinities for HLA-DQ8-CpepL11C and HLA-DQ8-HIPL11C with affinities of 11.8 ± 3.1 μM and 35.5 ± 1.5 μM, respectively (Fig. 3a, b and Supplementary Table 2). Accordingly, and in line with the cell stimulation assays, these affinity measurements revealed a spectrum of TCR cross-reactivity between the proinsulin C-peptide and HIP antigens presented by HLA-DQ8.

### TRBV5-1^+^ TCR-HLA-DQ8-HIP recognition

**Fig. 4:**
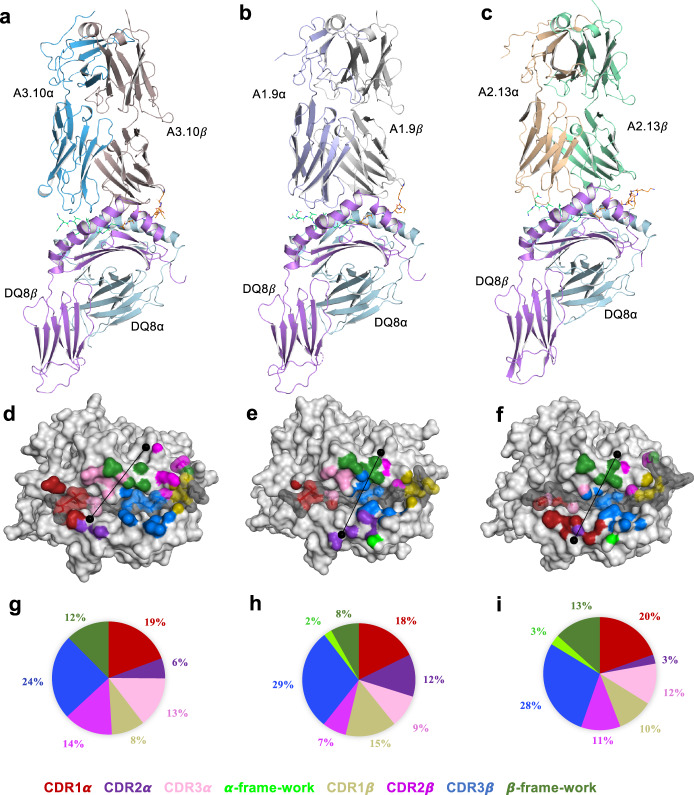
Structural overview of proinsulin C-peptide-responsive TCR-HLA-DQ8-HIPL11C ternary complexes. **a**–**c** Cartoon representation of TCR bound to HLA-DQ8-HIPL11C. The HLA-DQ8 α-chain is coloured in neon, and the HLA-DQ8 *β*-chain in light cyan. The TCRs are coloured as follows: **a** TCR A3.10 α-chain in sky-blue; *β*-chain in light brown; **b** TCR A1.9 α-chain in mauve; *β*-chain grey; **c** TCR A2.13 α-chain in wheat; *β*-chain in light green. The TCR CDR 1α, 2α, 3α loops, α -framework, CDR 1*β*, 2*β*, 3*β* loops and *β*-framework are coloured red, purple, pink, neon-green, gold, magenta, blue, and green, respectively. HIP peptide bound in HLA-DQ8 binding cleft is coloured in lime-green (C-pep portion), and orange (IAPP2 portion). **d**–**f** Atomic footprints of TCR A3.10, A1.9, and A2.13 on the surface of HLA-DQ8-HIPL11C, respectively. HLA-DQ8 α- and *β*-chain are coloured in light-grey, whilst HIPL11C is in dark grey. The CDR loops colours described above. TCR footprint colours are in accordance with the nearest TCR contact residues. The TCRs’ Vα and V*β* centre of mass position are shown as black dots, with the line approximating their docking angle. **g**–**i** The pie charts present the relative contribution of each CDR loop, α-, and *β*-framework of TCR A3.10, A.19 and A2.13 to the surface of HLA-DQ8-HIPL11C, respectively.

We next determined the ternary complexes of three TRBV5-1^+^ TCRs (A3.10, A1.9, and A2.13) bound to HLA-DQ8HIPL11C (Fig. 4a–c; Supplementary Table 3). As the A3.10, A1.9, and A2.13 TCRs used the same TRBV5-1 gene, this enabled us to examine the structural basis of the germline encoded TCR β-chain bias against the backdrop of differing CDR3β and TCR α-chain usage. The A3.10, A1.9, and A2.13 TCRs docked roughly similarly (docking angle range of 54^o^–69^o^) with a buried surface area (BSA) values ranging from 2050 Å^2^ to 2320 Å^2^. Here, the TCR β-chain played a dominant role at the interface (BSA $\approx$ 60%) (Fig. 4d–i), whereupon the CDR3β loop contributed most to the interaction (BSA $\approx$ 24–29 %), while the CDR1β, CDR2β and Vβ framework regions contributed roughly equally (BSA $\approx$ 8–15%)

**Fig. 5:**
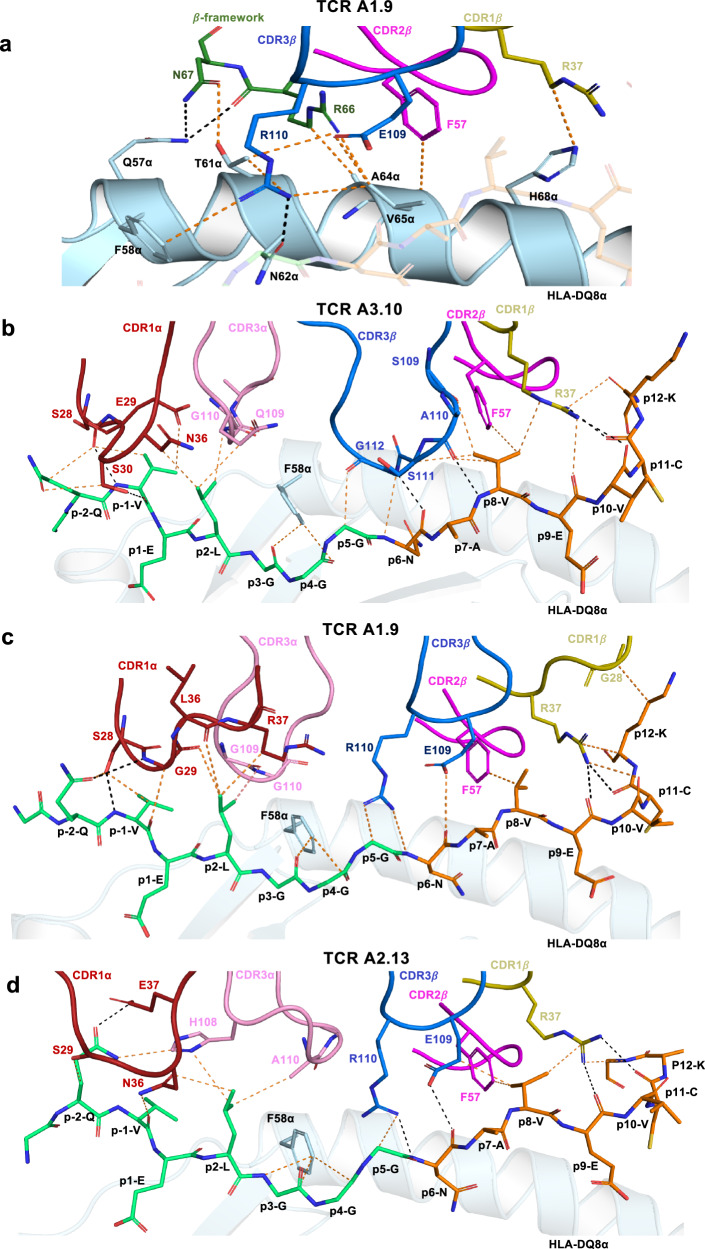
Interactions at the TRBV5-1+ TCR-HLA-DQ8-HIPL11C interface. **a** Conserved interactions between TRBV5-1+ TCR CDR*β* loops; *β*-framework and the HLA-DQ8 α-chain using TCR A1.9 as an example. The HLA-DQ8 α-chain is coloured in neon. Whilst the CDR1*β*, CDR2*β*, CDR3*β* loops and *β*-framework are in gold, magenta, blue, and green, respectively. **b**–**d** The CDR loops of A3.10, A1.9 and A2.13 TCRs make conserved contacts with the HIPL11C peptide. The CDR1α and CDR3α loops are coloured in red and pink, respectively, and the CDR*β* loops colours as described above. The HIPL11C peptide is coloured such that the C-peptide fragment is in lime-green and the IAPP2 fragment in orange. Hydrogen bonds (H-bonds) are denoted as black dashed lines, whilst vdW interactions are presented as orange dashed lines. Cartoon representations of the interactions between the CDR loop and HLA-DQ8-HIPL11C are shown with oxygen atoms in red and nitrogen atoms in blue.

In all three TCRs, the CDR1β and the CDR2β loops were similarly positioned atop HLA-DQ8-HIPL11C, making contacts with the peptide (discussed below) and the HLA-DQ8 molecule itself. Here, the contact between Arg37β from the CDR1β loop and HLA-DQ8 His68α was conserved. Phe57β from the CDR2β loop, which was situated adjacent to Arg37β, was positioned above Ala64α from HLA-DQ8 (Fig. 5a; Supplementary Tables 4–6). Moreover, the TCRβ-framework segment containing Arg66β and Asn67β interacted with the HLA-DQ8 α-chain, where Arg66β formed an H-bond with Gln57α and van der Waals (vdW) interactions with Thr61α, Ala64α, and Val65α whilst Asn67β interacted with Gln57α (Fig. 5a; Supplementary Tables 4–6).

The structure of the TRBV5-1^+^ T1D3 TCR in complex with HLA-DQ8 presenting an engineered insulin B:9-23 peptide (HLA-DQ8-insulin B:9-23mut; LVE***E*LYLV*A*GE*E***G*C*; mutated residues in italics, P1-P9 register in bold) has also been determined^24^. Many features of the three TRBV5-1^+^ TCR HLA-DQ8-HIPL11C structures presented here are conserved within the T1D3-HLA-DQ8-insulin B:9-23mut (T1D3-HLA-DQ8-8E9E11ss) peptide ternary structure, including a similar docking geometry and footprint on the peptide bound HLA-Class II (pHLA-II) (Supplementary Fig. 5a). Of note, many of the TRBV5-1 mediated interactions of the CDR1β Arg37β, CDR2β Phe57β and β-framework residues Arg66β and Asn67β, with the HLA-DQ8 α-chain were highly similar across all four structures. This is exemplified by the direct comparison of the interactions of the A3.10 and T1D3 TCRs with the HLA-DQ8 α-chain (Supplementary Fig. 5b–e). Accordingly, the driving force for biased *TRBV5-1* usage observed in the CD4+ T cell response to HLA-DQ8-HIP is due, in part, to a combination of conserved interactions within a focused region on the HLA-DQ8 α-chain.

### Interactions with the HIP peptide

HLA-DQ8 presents HIPL11C in a very similar manner to other HLA-DQ8 restricted epitopes^19,25–27^, where the residues P1-Glu and P9-Glu acted as anchor residues in the binding cleft of HLA-DQ8 (Supplementary Fig. 6a–c). Here, the peptide is stabilised in the groove by a salt-bridge forming between P1-Glu and Arg52α, while P9-Glu formed a salt bridge with Arg76α and a H-bond with Tyr37*β* from HLA-DQ8 (Supplementary Fig. 6a). Somewhat unusually, the HIP epitope contained three centrally located glycine residues, which demarcated the boundary between the spliced C-peptide and IAPP2 peptides (Supplementary Fig. 6b, c). The three A3.10, A1.9, and A2.13 TCRs bound the HIP peptide in a broadly similar manner, whereupon the TCR α- and β-chains engaged the C-peptide and IAPP2 regions of the HIP peptide, respectively (Fig. 5b–d). In all TCR ternary complexes, the CDR1α, CDR3α, CDR1β CDR2β and CDR3β loops were similarly positioned atop the HIP peptide (Figs. 4d–f and 5b–d). Within this framework, the interactions between the germline-encoded CDR1β and CDR2β loops and the C-terminal region of the HIP peptide were conserved (Fig. 5b–d), indicating that the TRBV5 bias is also attributable to IAPP2 peptide-mediated contacts. Differences in the TCR α-chain and CDR3β usage however resulted in some altered interatomic contacts at the TCR-HIP interface. Here we describe the A3.10 TCR-HIP contacts first, followed by highlighting salient aspects of the A1.9 and A2.13-HIP mediated contacts.

The CDR1α loop and CDR3α loop of A3.10 TCR interacted with the HIP peptide N-terminus. Here, Ser28α-Glu29α-Ser30α from the CDR1α loop formed a series of polar and vdW interactions with P-1-Val and P-2-Gln of the HIP peptide (Fig. 5b, Supplementary Table 4). P2-Leu is situated under the CDR1α and the CDR3α loops, forming extensive vdW interactions with the CDR1α (Asn36α) and the CDR3α (Gln109α, and Gly110α) loops (Fig. 5b, Supplementary Table 4). The A3.10 TCR β-chain interacted with the P5-P12 region of the HIP peptide. Here Arg37β from the CDR1β loop formed three points of contact at the C-terminal end of the peptide region from P8 to P12 (Fig. 5b). P8-Val positioned under the CDR1β, CDR2β, and CDR3β loops, formed extensive vdW interactions via Arg37β (CDR1β), Phe57β (CDR2β) and Ser109β-Ala110β-Ser111β from the CDR3β loop (Fig. 5b, Supplementary Table 4). The TCR A3.10 has a longer CDR3β loop comprising smaller side chains compared to the A1.9 and A2.13 TCRs. This permitted the CDR3*β* loop to dip into the HLA-DQ8 binding cleft and form interactions with positions P5-Gly, P6-Asn and P8-Val of the peptide (Fig. 5b, Supplementary Table 4).

In the A2.13 TCR ternary complex, CDR1α residues Ser29α and Glu37α, along with the CDR3α His108α, bound to P-2-Gln, seemed to lift the N-terminus of the peptide ~1.9 Å upwards compared to that observed for the A3.10 TCR-HIP interactions (5b, d, Supplementary Fig. 7a). Moreover, the CDR1α loop of the A2.13 TCR was shifted ~5.6 Å toward the HLA-DQ8 β-chain at the HIP N-terminus (Supplementary Fig. 7a). Similar to the A3.10 TCR, a vdW interaction was formed between CDR1α Asn36α and P2-Leu. Although there was an ~6.1 Å shift of the A2.13 TCR CDR3α loop apex compared to that of the A3.10 TCR, similar contacts to the P2-Leu were still enabled via CDR3α His108α and Ala110α (Figs. 5b, d, Supplementary Fig. 7a, b and Supplementary Tables 4 and 6). The A1.9 TCR CDR1α and the CDR3α loops were shifted approximately 7.3 Å and 5.4 Å, respectively, compared with that observed for the CDR1α and CDR3α loops of the A2.13 TCR (Supplementary Fig. 7a). This positioned them to make similar interactions to that observed for the A3.10 TCR (Fig. 5b, c). Of note, the CDR3β loop of the A2.13 TCR consisted of a similar motif (“LERE”) to that of A1.9 (“LERD”) (Supplementary Table 1) and displayed very similar interactions as observed for A1.9 TCR, including the interactions of Arg110β with P5-Gly, and Glu109β (Fig. 5c, d, Supplementary Tables 5, 6).

We have used a disulfide bridge between the HIP at P11 and HLA-DQ8αI72C to stabilise the pHLA-II complex. Hence, it is important to note that in all three TCR-HLA-DQ8-HIPL11C ternary complexes, the interactions between the TCRs and the peptide at P11 involved the peptide main chain and thus the disulfide bridge plays no role in an interaction network with the TCR at this position (Supplementary Tables 4–6). This was in keeping with the surface plasmon resonance^23^ results showing no significant difference between the affinity of these TCRs for HLA-DQ8-HIPL11C versus HLA-DQ8-HIP (Supplementary Table 2, Fig. 3, Supplementary Fig. 3).

Accordingly, we provide a portrait of how three TCRs, exhibiting varied TCR gene usage, can engage the common HLA-DQ8-HIPL11C complex. The structural data provide a general mechanism to which this HIP peptide is engaged, namely the TRBV5-1^+^ TCR α-chain and TCR-β-chain interacts with the C-peptide and IAPP2 moieties, respectively, and as such appear to adopt a ‘polarised’ modality for spliced peptide recognition.

### Fine specificity toward HIP determinant

We next investigated the fine specificity of three HIP reactive T cell clones, including A3.10, A1.9 and a clone A5.5 with a mild preference for the HIP peptide over the C-peptide. We used a panel of HIP peptides with single alanine substitutions at each position (P-3 to P12) except P7-Ala. Substitution of P-1-Val, P1-Glu, P2-Leu, P3-Gly, P8-Val, and P9-Glu to alanine significantly or completely led to a loss in stimulatory activity for all three T cell clones, which was consistent with the similar mode of interaction of the TRBV5 + TCRs with HLA-DQ8-HIPL11C (Supplementary Fig. 8). Moreover, the impact of these mutations underscores the importance of both the C-peptide and IAAP2 segments within the hybrid spliced peptide in mediating key contacts with the TCRs. Substitution of P1-Glu, or P9-Glu would destabilise the HIP peptide binding by HLA-DQ8, thus preventing presentation of these peptides in HLA-DQ8, leading to a corresponding lack of T cell recognition. (Supplementary Fig. 8). P-1-Val, P2-Leu, P3-Gly, and P8-Val represented TCR contact points, and thus mutation to alanine directly impacted T cell recognition. That the A3.10 T cell clone is 100 fold more sensitive to HIP than the proinsulin C-peptide is indicative that the HIP is not merely a mimotope of the proinsulin peptide. Indeed, the HIP specific P8-Val mediates numerous interactions with the A3.10 TCR (Supplementary Table 4) which turn out to be key contacts as mutation to P8-Val abolishes HIP recognition by the A3.10 T cell clone (Supplementary Fig. 8).

There were also some differences observed in the sensitivity to particular mutated residues. Namely, stimulation of the clone A5.5 was abolished with alanine mutation of P-2-Gln, whereas clones A3.10 and A1.9 were less severely affected and, conversely, alanine mutation to P5-Gly abolished stimulation of clones A3.10 and A1.9 and had no effect on clone A5.5. Clones A3.10 and A5.5 were both unaffected by mutation to P6-Asn whereas the response of clone A1.9 was abolished by alanine mutation at this position (Supplementary Fig. 8). Given the TCR A3.10 and A1.9 contacts with P10-Val, P11-Cys and P12-Lys were to main chains (Fig. 5b, c and Supplementary Tables 4, 5), it is not surprising that mutation of these to alanine had no significant effect on TCR recognition and further supports the SPR data indicating that the introduced disulfide bridge between P11-Cys and HLA-DQ8αI72C does not significantly affect TCR recognition.

Hence, whilst all three clones tested had several residues in common that were essential for epitope recognition, each clone had unique differences in their sensitivity to alteration in the HIP peptide. This relates to the distinct TCR gene usage from each T cell clone manifesting in differing interatomic contacts with HLA-DQ8-HIP, which is also reflected in their differential sensitivity to the HIP peptide and levels of TCR cross-reactivity (Fig. 2).

### Identification of HLA-DQ8-HIPL11C reactive CD4+ T cells in PBMCs from patients with T1D

The CD4+ T cell clones and their respective TCRs used for the cellular, structural and biophysical analysis outlined above were isolated from the pancreas of a deceased T1D patient. We wanted to determine whether such clones were also present in the circulation of patients with T1D. Hence, we stimulated CFSE-labelled PBMCs isolated from three recent onset T1D patients (Patient ET600, ET650 and ET672; (Supplementary Table 7) using the HIP peptide. HIP reactive cells were subsequently single cell sorted by FACS using an HLA-DQ8-HIPL11C tetramer (Supplementary Fig. 9 representative example of ET650) and the TCR αβ gene sequences of these clones were determined (Table 1). Given we used an in vitro expansion process, not all HIP reactive clones will necessarily be stimulated or at the same rate under the conditions used and the frequency of any responding T cell clones isolated from PBMCs will not be indicative of their original frequency in the starting population. Patient ET650 had HLA-DQ8-HIPL11C reactive clones that shared the same *TRAV26-1*-*TRBV5-1* gene usage observed in three of the five clones isolated from pancreatic islets used here, namely the A2.13, A5.5, and A5.8 TCRs (Supplementary Table 1) TRAV26-1 usage was also represented in two HLA-DQ8-HIPL11C reactive clones, one of which was expanded, from Patient ET600 along with one clone using *TRBV5-1* (Table 1). Remarkably, in Patient ET650, the most highly represented clone, ET650-1, had an almost identical αβ TCR usage to that of the A5.5 TCR with only one residue difference observed between the two CDR3β regions (Table 1 and Supplementary Table 1). Such close homology between two TCRs restricted to the same antigen in two individuals, along with the biased usage of the *TRAV26-1* and *TRBV5-1* genes separately or paired in three different T1D patients, is indicative of a public response elicited to the spliced HIP peptide when bound to HLA-DQ8^28^. Although we did not observe the TRAV26-1/TRBV5-1 biased usage in Patient ET672, unlike the two other patients that had several different HLA-DQ8-HIPL11C responsive clones, this patient had a virtual single clonal expansion with 18/19 sequences having the same TCR gene (Table 1). Using SPR, we confirmed the pHLA-II restriction of representative TCRs ET650-2, ET650-4, ET650-5, and ET672-1 by determining their affinity to HLA-DQ8-CpepL11C and HLA-DQ8-HIPL11C (Supplementary Fig. 10) as well as HLA-DQ8-HIPL11C tetramer specific staining of SKW-3 T cells transduced with these TCRs (Supplementary Fig. 11). Given the specific tetramer staining of ET650-2 clones ET650-5 was weak, we also performed activation assays using SKW-3 T cells transduced with these TCRs and confirmed their restriction to HLA-DQ8-HIP (Supplementary Fig. 1b, d). All, except SKW-3-ET650-5, were cross-reactive with HLA-DQ8-C-peptide, which corresponded with the TCR affinity data (Supplementary Figs. 1b, d, 10).

**Table 1:** TCR gene usage of CD4 + HLA-DQ8-HIPL11C tetramer^+^ T cells in T1D patients ET600, ET650, and ET672 PBMC.

Clone	TRAV	TRAJ	CDR1α	CDR2α	CDR3α	TRBV	TRBJ	TRBD	CDR1β	CDR2β	CDR3β	No. clone
ET600-1	26-1	33*01	TISGNEY	GLKN	CIVRDSNYQLIW	29-1	1-2*01	1*01	SQVTM	ANQGSEA	CSANRDGGGYTF	7
ET600-2	8-3	22*01	YGATPY	YFSGDTL	CAVGGTSGSARQLTF	10-3	1-2*01	2*01	ENHRY	SYGVKD	CAISEGERGSNYGYTF	6
ET600-3	8-3	27*01	YGATPY	YFSGDTL	CAVGFNAGKSTF	5-1	1-1*01	1*01	SGHRS	YFSETQ	CASSLGPTGTNTEAFF	1
ET600-4	14/DV4	39*01	TSDPSYG	QGSYDQQ	CAMRPNNAGNMLTF	15	1-5*01	1*01	LNHNV	YYDKDF	CATSRGNQGPNQPQHF	1
ET600-5	26-1	39*01	TISGNEY	GLKN	CIVRVNNAGNMLTF	28	1-2*01	2*01	MDHEN	SYDVKM	CASSRLDNGYTF	1
ET600-6	12-2	15*01	DRGSQS	IYSNG	CAVNQAGTALIF	11-1	2-1*01	1*01	SGHAT	FQDESV	CASSWAYNEQFF	1
ET600-7	3	6*01	VSGNPY	YITGDNLV	CAVRDGGSYIPTF	7-8	2-7*01	1*01	SGHVS	FQNEAQ	CASSLLPDRGHEQYF	1
ET600-8	19	9*01	TRDTTYY	RNSFDEQ	CALSEVNTGGFKTIF	10-3	2-6*01	1*01	ENHRY	SYGVKD	CAISLQGSRSGANVLTF	1
ET650-1	26-1	54*01	TISGNEY	GLKN	CIVRVEIQGAQKLVF	5-1	2-5*01	1*01	SGHRS	YFSETQ	CASSLGPGLRETQYF	4
ET650-2	26-1	39*01	TISGNEY	GLKN	CIVRVGYNAGNMLTF	20-1	1-5*01	2*01	DFQETT	SNEGSKA	CSAIAGPNQPQHF	3
ET650-3	26-1	42*01	TISGNEY	GLKN	CIVRPQKGGSQGNLIF	3-1	2-3*01		LGHDT	YNNKEL	CASSHHSTDTQYF	2
ET650-4	26-1	42*01	TISGNEY	GLKN	CIVRVAIEGSQGNLIF	5-1	1-3*01	1*01	SGHRS	YFSETQ	CASSLRRGDTIYF	1
ET650-5	26-1	9*01	TISGNEY	GLKN	CIVRLQSGGFKTIF	20-1	1-2*01	1*01	DFQETT	SNEGSKA	CSAYSPGDRDFSNYGYTF	1
ET650-6	39	54*01	TTSDR	LLSNGAV	CAVDIQGAQKLVF	7-8	2-3*01	1*01	SGHVS	FQNEAQ	CASSLRTAISRTDTQYF	1
ET650-7	30	48*01	KALYS	LLKGGEQ	CGTLAFGNEKLTF	19	1-6*01	2*01	LNHDA	SQIVND	CASHRGKGNSPLHF	1
ET650-8	35	49*01	SIFNT	LYKAGEL	CAGQLWNTGNQFYF	11-1	2-3*01	2*01	SGHAT	FQDESV	SHLRGASTDTQ	1
ET672-1	12-2	48*01	DRGSQS	IYSNGD	CAVNHGNEKLTF	18	1-1*01	1*01	KGHSH	LQKENI	CASSPWEGRMDTEAFF	18
ET672-2	12-1	37*02	NSASQS	VYSSGN	CVVVSNTGKLIF	23-1	2-1*01	2*01	KGHTF	FQNEQV	CASSQWVSGEDEQFF	1

Accordingly, we show that HLA-DQ8-HIPL11C reactive CD4+ T cell clones are present in the periphery of recently diagnosed T1D patients and we provide evidence that this antigen generates public clonotypes within the TCR repertoire of these individuals.

## Discussion

The HLA Class II locus has the strongest genetic association for susceptibility to T cell mediated autoimmune diseases, including the HLA-DQ8-T1D axis^29^. Presently, several mechanisms are proposed to explain this association, including alternate docking of TCRs to pHLA-II, low-TCR-pHLA-affinity interactions mediating thymic escape, TCR stabilisation of low affinity pHLA-II complexes, altered peptide binding registers to HLA-II, HLA stability and level of cell surface expression, and PTM of peptides that generate neoantigens^30^. In relation to PTMs and T cell mediated autoimmunity, current examples include deamidation of glutamine residues in coeliac disease^19,25,26,31,32^, citrullination of arginine residues in rheumatoid arthritis^33,34^, and disulfide bond formation in T1D^35^. Peptide-splicing, where there is the fusion of two non-contiguous peptide fragments, has been associated with T1D^5–7,10,18,36,37^. Data suggests these hybrid peptides are generated by cathepsin L mediated transpeptidation after secretory granules containing pancreatic proteins fuse with lysosomes in pancreatic β-cells^18^. Here, we have shown how naturally occurring spliced peptides are commonly presented by HLA-DQ8, described the T cell repertoire responding to HLA-DQ8 presenting a HIP determinant, and provided mechanistic insight into how TCRs interact with this HLA-DQ8-HIP.

We used in an in vitro cultivated human APC to gain an understanding of the frequency of spliced peptides in the normal MHC-II peptidome. The HLA-DQ8 peptide binding motifs we generated showed some divergence, particularly at the N-terminus of putative spliced peptides, compared to that observed by Suri et al.^20^. Given differences in species of the antigen presenting cells, human in our study and mouse in Suri et al.^20^ (NOD.DQ8), as well as the total number of peptides in the datasets used to generate the motifs (206 (Suri et al.^20^) vs 5441 (this study)) these subtle differences may be anticipated. Here, we established that, in an in vitro cultivated human APC, spliced peptides represent ~25% of all PTMs. Supporting our observations is the report examining the MHC-II peptidome in the pancreatic islets and peripheral lymph nodes of NOD mice that identified a proinsulin C-peptide-IAPP2 (LQTLAL–NAARD) HIP that had T cell autoreactivity^36^. Although only one HIP was found in the MHC-II peptidome of APCs in this study, this may be due to the challenging nature of the samples examined and low abundance peptides may have been missed. Indeed, HIPs may be presented at relatively low abundance compared to the 240 peptides identified that were derived from mainly highly abundant source proteins^36^.

The length and composition of peptide sequences flanking the 9-mer core epitope bound to MHC-II has been shown to affect TCR antigen recognition^38^. Indeed, our structural data showed multiple interactions of the all three TCRs with the peptide outside the antigen binding cleft, particularly main chain, as opposed to side-chain, interactions at P10, P11 and P12. Alanine mutation to positions P-3, P10, P11 and P12 of the HIP peptide had no effect on T cell stimulation by the CD4 T cell clones tested, which confirms the structural observations that, while the length of peptides clearly play a role in the TCR recognition presented here, this was generally independent of the composition of the of flanking residues.

T cell stimulation assays showed that each of five original pancreatic islet CD4+ T cell clones had differential reactivity to the full length proinsulin C-peptide and HIP epitopes, despite all five sharing the same *TRBV5-1* gene usage. Previous work on the TCR gene usage within the proinsulin C-peptide responsive CD4+ T cell repertoire isolated from the pancreatic islets of a deceased T1D patient, revealed that a biased T cell response was evident^4^. Namely, the *TRAV26-1* and *TRBV5-1* genes were over-represented in the CD4+ T cell repertoire of this patient. The three TRBV5-1^+^ TCR-HLA-DQ8-HIPL11C ternary complexes revealed that the biased *TRBV5-*1 gene usage is due, in part, to a combination of conserved interactions of the CDR1β-Arg37β, CDR2β-Phe57β and β-framework (Arg66β, Asn67β) residues interacting within a focused region of the HLA-DQ8 α-chain.

Yang et al.^39^ described a TRBV5-1^+^ HLA-DQ8 restricted T cell clone isolated by insulin B:9-23 peptide (B:9-23 SHLVEALYLVCGERG) tetramer staining^39^. Given the poor affinity of this peptide for HLA-DQ8, and a corresponding low reactivity of the TRBV5-1^+^ T1D3 T cell clone to this epitope, a series of mutations were engineered to promote the production of a stable HLA-DQ8-peptide complex. The set of mutations comprised P1AtoE, P6CtoA and P9RtoE and P11FtoC (LVE***E*LYLV*A*GE*E***G*C*; mutated residues in italics, P1-P9 register in bold)^22,24,39^. This engineered epitope was used as a model put forward for how a hybrid insulin peptide could generate a higher affinity epitope that is subsequently recognised as a neoantigen in T1D patients^22,24^. This engineered epitope was indeed recognised by the TRBV5-1^+^ T1D3 TCR and the TCR-pHLA-II ternary complex structure showed notable similarity with the three TRBV5-1^+^ HIP TCR ternary structures^24^, including the docking geometry and the TRBV5-1 mediated TCR footprint on the pHLA-II.

From the available database of TCR-peptide-MHC structures^40^, the TCR α−chain and β-chain are, with two notable exceptions^41,42^, positioned over the α2 and α1 helix of the MHC-I molecule (or, for MHC-II, the β-chain and α-chain). Often TCRs are situated centrally atop the MHC molecule, with the TCR β-chain and α-chain binding to the N- and C-terminal regions of the bound peptide. So, our observations surrounding spliced peptide recognition could merely be reflective of this general consensus. However, there are also numerous examples of TCRs adopting N-terminal or C-terminal shifted footprints on the MHC^43,44^. Accordingly, a priori it was unclear how TCRs would interact with non-germline encoded peptide bound to the MHC. We show in all three HIP TCR-HLA-DQ8 ternary structures, the interactions of the α- and β-chains with the HIP peptide were segregated according to the donor segments of the hybrid peptide. That is, the TCR α- and β-chains only contacted the proinsulin C-peptide and the IAPP2 portion of the HIP, respectively. Our findings suggest a concept of a ‘polarised’ mechanism of HIP peptide recognition by TRBV5 + TCRs. This hypothesis will be examined further, by direct comparison of CD4+ T cells that respond to HIP and C-peptide from peripheral blood of T1D patients.

## Methods

### Isolation of peptides bound to HLA-DQ-8 and mass spectrometry

9033 Epstein-Barr virus (EBV)-transformed B-lymphoblastoid cells (BLCL; HLA-DQ8 + : HLA-DQA1*03, HLA-DQB1*03:02) pellets were frozen in liquid N_2_ and stored at −80 °C. Peptides presented by HLA-DQ8 were extracted from immunoprecipitated HLA-DQ8 molecules and analysed using mass spectrometry as follows. Cell pellets were ground using a Retsch mixer mill (MM400) (Retsch) and lysed in 0.5% (v/v) NP-40, 50 mM Tris pH 8.0, 150 mM NaCl, and protease inhibitor cocktail (Roche cOmplete Protease Inhibitor) and HLA-DQ-peptide complexes isolated by immunoaffinity purification using protein A-crosslinked anti-DQ antibody (10 mg per sample, clone SPV-L3). HLA-peptide complexes were dissociated by addition of 10% (v/v) acetic acid and peptides separated from HLA-DQα and DQβ-chains by RP-HPLC fraction using an Äkta Ettan (GE Healthcare) HPLC. Peptides and HLA proteins were separated using a Chromolith SpeedROD (RPC18 end-capped, 100 × 4.6 mm) column (Merck) and a mobile phase incorporating an increasing gradient mixture of buffer A (0.1% v/v trifluoroacetic acid (TFA) in water) and buffer B (80% v/v acetonitrile and 0.1% v/v TFA in water). Peptide containing fractions were pooled using a concatenating scheme of every seventh fraction, dried down using a centrifugal concentrator (Labconco) and resuspended in 20 µl of mass spectrometry buffer A (0.1% v/v formic acid (FA) in water).

For LC-MS/MS a Dionex UltiMate 3000 RSLCnano UHPLC was used to load samples onto an Acclaim PepMap C18 100 trap column (100 µm × 2 cm; Thermo Fisher Scientific), followed by separation of the analytes using an Acclaim PepMap RSLC C18 analytical column (75 µm × 50 cm; Thermo Fisher Scientific). The separation of peptides was achieved by a linear gradient of 0–80% ACN/0.1% FA concentration with a 250 nL/min flow rate for 158 min and analysed with an Orbitrap Fusion Tribrid mass spectrometer (Thermo Fisher Scientific) using Orbitrap Tribrid MS Series Instrument Control Software Version 3.3 (ThermoScientific, San Jose, CA, USA) and a data-dependent acquisition strategy with the following settings:

All MS spectra (MS1) profiles were recorded from full ion scan mode 375–1800 m/z in the Orbitrap at 120,000 resolution with automatic gain control (AGC) target of 400,000 and dynamic exclusion of 15 s. The top 12 precursor ions were selected using top speed mode at a cycle time of 2 s. For MS/MS, a decision tree was made which helped in selecting peptides of charge state +1 and +2 to +6 separately. For single charged analytes ions falling within the range of m/z 800–1800 were selected. For +2 to +6 m/z s no such parameter was set. The ctrap was loaded with a target of 200,000 ions with an accumulation time of 120 ms and isolation width of 1.2 amu. Normalised collision energy was set to 32 (high energy collisional dissociation (HCD)) and fragments were analysed in the Orbitrap at 30,000 resolution.

Data were analysed using PEAKS Studio v. Xplus (Bioinformatics Solutions Inc) with the following settings: parent mass error tolerance of 10 ppm; fragment mass error tolerance of 0.02 Da; no enzyme cleavage; variable modifications of oxidation (M). Data were searched against the human proteome (Uniprot, November 2018). PEAKS PTM, was subsequently performed in which unassigned spectra were searched against the human proteome (Uniprot, November 2018) database by including 55 common modifications with a FDR cut-off of 1% applied. The top 20 high confidence de novo sequenced candidates without any linear peptide match were further interrogated with the “Hybrid finder” algorithm^11^ and the identified *cis*- and *trans*-spliced candidate sequences added back to the original database. A “Multi-Run Search with Denovo Only Spectra” was performed by using the combined database. Linear and spliced peptides in this search were extracted at 1% FDR to create the final list of identified peptides.

### Synthetic peptides

Peptides were synthesised by GL Biochem using Fmoc chemistry. Peptides were purified by RP-HPLC to at least 85% purity and lyophilised. Peptides were reconstituted in 40% acetonitrile, 0.5% acetic acid, water to a concentration of 5 mM, aliquoted and stored at −80 ^o^C.

### T cell assays

For primary T1D CD4+ T cell clones, cells were thawed and used directly in functional assays. For T cell stimulation assays, cloned CD4+ T cells were incubated with BLCL KJ (; HLA-DQ8 + : HLA-DQA1*03:01, HLA-DQB1*03:02), as APCs, together with synthetic peptides. Cloned CD4+ T cells (5 ×10^4^/well) were cultured with APCs (5 ×10^4^/well) with and without peptides as indicated in the figures. For the alanine scan experiments, each peptide was added to a final concentration of 10 μM. T cell response to antigen was measured as interferon gamma (IFNγ) secretion into the culture media. The IFNγ concentration in the culture media was determined by ELISA (Biolegend).

For TCR transduced SKW-3 T cell lines (SKW-3-TCR), response to antigen was determined using flow cytometry to assess the level of upregulation of the T cell activation marker CD69. One hundred thousand BLCL 9031 cells (HLA-DQ8 + : HLA-DQA*03:01:01, HLA-DQB1*03:02) as APCs were incubated at 37 ^o^C, 5% CO_2_ for 24 h with increasing concentrations of synthesised HIP (GQVELGGGNAVEVLK; GLBiochem) or native C-peptide (GQVELGGGPGAGSLQ; GLBiochem) or 50 μg DQ8-glia-α1 (control) in 96 well round-bottomed plates. To confirm HLA-DQ8-restriction, 2 μg/ml (SKW-3-ET672-1) or 50 μg/ml (all other SKW-3-TCR cell lines) C-peptide or HIP pulsed BLCL 9031 cells were blocked with a final concentration of 20 μg/ml anti-HLA-DQ monoclonal antibody (clone SPV-L3) for 3 h before stimulation (i.e. before the addition of SKW-3-TCR cell lines). T-cell activation assay were performed by mixing 1 × 10^5^ SKW-3-TCR cells, SKW-3 parental cells (control) or SP3.4 (HLA-DQ8-glia-α1 restricted T cell line; control) cells with the peptide-pulsed BLCL 9031 cells or with 5 μl anti-CD3/CD28 dynabeads (Thermo Fisher Scientific) (control) at 37 ^o^C, 5% CO_2_ for 20 h before antibody staining. The activated induced SKW-3-TCR T cells were washed twice with FACS buffer (Phosphate buffered saline, 10% Fetal Calf Serum (FCS; Merck)) (350 g, 5 min), and stained a mixture of 1:100 diluted V450 mouse anti-human CD3 (clone UCHT1, BD Biosciences) and APC Mouse Anti-Human CD69 (Clone FN50, BD Biosciences) for 1 h on ice in the dark. The samples were washed twice with PBS buffer (350 g, 5 min) to remove excess antibodies. Afterward, the samples were stained for live/dead cells with Zombie NIR (Biolegend) for 30 min at RT in the dark before being washed 6 times with FACS buffer (350 g, 5 min). Before data acquisition, the samples were resuspended in 100 μl of FACS buffer and analysed via flow cytometry (LSR II; BD Biosciences; BD FACSDiva-8.0.1 software). Collected data were analysed using Flowjo v10.6.0 (FlowJo), and then Prism 9 (GraphPad Software). The flow cytometry gating strategy for the cellular activation assay- (Gate 1): SKW-3-TCR lymphocytes; 10,000 events from single cells (Gate 2) were acquired from SKW-3-TCR lymphocytes; from Gate 2, live cells were isolated (Gate 3); The double GFP + RFP + cells (Gate 4) were acquired from the live cell population and then displayed as pseudocolor plots or histograms of CD3 or CD69 versus forward scatter (Fcs-H) from the cells with positive GFP + RFP + . Using these settings, the median fluorescence intensity (MFI) corresponding to the relative level of CD69 expressed on the SKW-3-TCR.TCR cell lines was determined using Flowjo v10.6.0 software. The MFI of CD69 were analysed by using Prism 9 (GraphPad Software). Three to six independent experiments were conducted, and all sample were performed in duplicates. Statistical significance was determined using P-values generated by t-test or one-way ANOVA multiple comparison of the MFI of no-peptide-stimulated SKW-3-TCR T cells versa the MFI of peptide-stimulated SKW-3-TCR T cells.

### Analysis of HLA-DQ8-HIPL11C restricted T cell repertoires

Ethical approval was provided by the Southern Health Research Ethics Committee (HREC Reference number: 12185B) and the Royal Children’s Hospital Research Ethics committee (SSA No.SSA/36346, RCH HREC No. 36346). All participants gave informed consent according to the National Health and Medical Research Council’s (NHMRC’s) National Statement on Ethical Conduct in Human Research and HRECs guidelines. If participants were under 18 years of age informed consent was obtained from their parents or legally authorised representatives. Participants with T1D within 100 days of diagnosis were recruited. T1D was diagnosed according to American Diabetes Association criteria. Peripheral blood mononuclear cells (PBMC) were isolated over Ficoll and labelled with CFSE (5,6- carboxylfluorescein diacetate succinimidyl ester) as described previously^45^. Briefly, PBMC were labelled with 0.01 μM CFSE (Thermo Fisher Scientific) and cultured (0.5 × 10^6^ cells in 0.5 ml were cultured in sterile 5.0 ml tubes. CFSE-labelled PBMC were cultured with either: no antigen, C-peptide-IAPP2 HIP (10 μM), or tetanus toxoid (10 LfU/ml). After 7 days of culture the cells were washed in PBS and stained on ice with Alexa Fluor 647 anti-human CD4 (clone OKT4 WEHI Antibody Facility), used at 0.5 μg/ml) and HLA-DQ8-HIPL11C tetramer or HLA-DQ8-glia-α1 tetramer (PE labelled; 1 μg/100 μl/sample). Optimal compensation and gain settings for the flow cytometer were determined for each experiment based on unstained and single stained samples. Viable (propidium iodide negative) CD4 + cells were gated and analysed for CFSE dilution and tetramer staining. Individual HLA-DQ8-HIPL11C tetramer specific CD4+ T cells were single cell sorted by FACS on BD FACSAria III, BD InFlux, and BD FACSAria Fusion (BD Biosciences) instruments using BD FACSDiva-8.0.1 (BD Biosciences) software for aquisition. The mRNA from single cells was reverse transcribed, and a multiplexed nested PCR strategy was used to amplify cDNA encoding CDR3α and CDR3ß regions using a panel of nested *TRAV*- and *TRBV*-specific oligonucleotide primers^46^ (Supplementary Table 8). PCR products were then purified and sequenced on an ABI 3730 DNA Sequencer and *TRAV* and *TRBV* gene usage determined using the IMGT database search engine IMGT/V-QUEST (http://www.imgt.org/IMGT_vquest/vquest?livret=0&Option=humanTcR)^47^.

### TCR transduction of T cell lines

Genes encoding HLA-DQ8-C-pep/HIP restricted TCR α- and β-chains (Twist Bioscience) were cloned into the lentivirus vectors (Biosettia) pLV-EF1α-MCS-IRES-GFP and pLV-EF1α-MCS-IRES-RFP, respectively, to generate pLV-EF1α-TCRα-IRES-GFP and pLV-EF1α-TCRβ-IRES-RFP. A mixture of 5 μg (1:1 ratio) of pLV-EF1α-TCRα-IRES-GFP and pLV-EF1α-TCRβ-IRES-RFP plasmids together with lentiviral packaging plasmids (Addgene: 1.5 μg pMD2.G, 2.5 μg, pMDLg/pRRE, 1 μg pRSV-REV) and 40 μl Fugene6 (Promega) was incubated in 470 μl OPTI-MEM I (1x) (Thermo Fisher Scientific) media at RT for 30 min. Approximately, 2 ×10^6^ HEK293T cells were transfected with Fugene-DNA mix and cultured in 10 ml fresh RF10 media: RPMI-1640 (Thermo Fisher Scientific) supplemented with 50 IU ml−1 penicillin, 50 μg ml−1 streptomycin, 2 mM glutamine, 1× nonessential amino-acids, 1 mM pyruvate, 10 mM HEPES (all from Thermo Fisher Scientific), 50 μM 2-mercaptoethanol (Merck) and 10% FCS (Merck). The transfected HEK293T cells were incubated at 37 ^o^C for 24 h before changing into fresh RF10 media. Transfection supernatants containing recombinant lentivirus was harvested at 48, 72, and 96 h post transfection in individual harvests which was then used for SKW-3-TCR transduction.

Four hundred thousand SKW-3 cells (TCR deficient; German Collection of Microorganisms and Cell Cultures) were serially transduced with 3 ml of filtered 48 h, 72 h and 96 h post-transfected lentivirus supernatants with the addition of 3 μg Polybrene (Merck) in a 6 well-plate, with 24 h between the addition of each lentivirus supernatant. Lentiviral transduced SKW-3 cells were washed 4 times with FACS buffer to remove remaining lentivirus in cultures.

The transduced SKW-3 cells were stained with BUV395 mouse anti-human CD3 (clone UCHT1, BD Biosciences) and APC mouse anti-human CD4 (clone RPA-T4, BD Biosciences) antibodies on ice for 1 h before washing 3 times with FACS buffer. The transduced SKW-3 cells were then live/dead stained with DAPI (BD Biosciences) for 15 min on ice before single cell sorting GFP + RFP + CD3 + CD4 + cells on a BD FACSAria Fusion apparatus (BD Biosciences) using BD FACSDiva-8.0.1 software (BD Biosciences). The single cell sorted TCR transduced clones were expanded in RF10.

TCR stable SKW-3 cells (~3 ×10^5^ in 200 μl) were stained with HLA-DQ8-HIPL11C tetramer or HLA-DQ8-glia-α1 tetramer (both APC labelled, BD Biosciences; used at 2 μg/200 μl) for 1 h at RT in the dark before staining with V450 mouse anti-human CD3 (clone UCHT1, cat. no. 561812, BD Biosciences) on ice for 1 h. The stained TCR stable cells were washed 3 times with FACS buffer before analysing on a Fortessa X20C apparatus (BD Biosciences) using BD FACSDiva-8.0.1 software (BD Biosciences). Collected data were analysed using FlowJo v10.6.0 software (FlowJo).

### TCR expression, refolding and purification

Soluble extracellular domains with an engineered interchain disulfide bond of the αβTCRs were produced by refolding of *E. coli* expressed inclusion body preparations as previously described^26^. Briefly, gene fragments (IDT) encoding the TCR α- or β-chains (A3.10, A1.9, A2.13, A5.5, A5.8, ET650-2, ET650-4, ET650-5, and ET672-1) were separately cloned into the pET30 *E. coli* expression vector. TCRs were expressed as inclusion bodies in the BL21(DE3) *E. coli* strain. TCR A1.9 was expressed with a complementary fos/jun leucine zipper tag attached to the C-terminal ends of the α- and β-chains, respectively, with thrombin cleavage sites between the α- and β-chains and fos/jun leucine zipper included. The proteins were isolated from inclusion bodies and resuspended in 6 M Guanidine hydrochloride buffer (6 M Guanidine hydrochloride, 20 mM Tris-HCl pH 8, 0.5 mM Na-EDTA, and 1 mM DTT). TCRs were refolded by rapid dilution of 120 mg IBs of α- and β-chains in a solution containing 5 M Urea Refolding Buffer (5 M Urea, 100 mM Tris-HCl pH 8, 480 mM L-arginine-HCl, 2 mM Na-EDTA, 0.2 mM PMSF, 0.5 mM Oxidised Glutathione, 5 mM Reduced Glutathione) for 72 h at 4 ^o^C. The refolding solution was then dialysed three-time against 15 L of dialysis buffer (10 mM Tris-HCl pH 8.0). The refolded TCRs were purified via gravity flow on DE52 AIE Cellulose columns (Cytiva) and eluted with TBS500 (10 mM Tris pH 8.0, 500 mM NaCl). TCRs were further purified using size exclusion (HiLoad 16/600 Superdex 200 pg column; Cytiva), hydrophobic interaction (HiTrapTM Phenyl HP column; Cytiva) and anion-exchange (HiTrapTM Q HP column; Cytiva) chromatography. For TCR A1.9, thrombin (Sigma) was used to cleave the zipper and purified on a HiTrap Q HP anion-exchange column (Cytiva).

### HLA-DQ8-peptide expression and purification

The regions encoding the extracellular domains of the HLA-DQ8 α- and β-chains (HLA-DQA*03:01 HLA-DQB1* 03:02) (IDT) were cloned into pZIP3 vector^25,26,31^ which contains fos/jun leucine zipper. In constructs with peptides containing P11C mutations, the HLA- DQA*03:01 gene encoded an I72C mutation for covalent bond formation with the peptide. Gene segments (IDT) encoding the CpepL11C (GQVELGGGPGAGS**C**Q), HIP (proins C-peptide–IAPP2 HIP: GQVELGGG*NAVEVLK*) and HIPL11C (GQVELGGG*NAVEV***C***K*) (binding register underlined, IAPP2 sequence in *italics*), class II-associated invariant chain peptide (CLIP) (ATPLLMQALPMGA) and HLA-DQ8-glia-α1 (PSGEGSFQPSQENPQ) peptides were inserted between sequences encoding the AcMNPV baculovirus gp67 signal peptide and a flexible Factor Xa cleavable linker (GSGGSIEGRGGSG) attached to the N-terminus of the HLA-DQ8-β-chain. The c-termini of the α- and β-chains contained an enterokinase cleavable Fos and Jun leucine zipper, respectively, for correct chain pairing. Following the Jun leucine zipper, the c-terminus of the β-chain contained a BirA *E. coli* biotin ligase recognition sequence (GLNDIFEAQKIEWHE) for biotinylation followed by a polyhistidine-tag for immobilised metal affinity chromatography (IMAC). The HLA-DQ8-peptide monomers were produced essentially as previously described^32^. Briefly, the supernatant of High Five insect cell (*Trichoplusia ni* BTI-TN-5B1-4 cells; Thermo Fisher Scientific) cultures infected with recombinant baculovirus (Bac-to-Bac, Thermo Fisher Scientific) was harvested after 72 h at 21 ^o^C and concentrated and buffer exchanged by tangential flow filtration (TFF) (Cogent M1, Merck). HLA-DQ8-peptide monomers were isolated by purification via IMAC; (Ni-NTA Superflow; Qiagen) followed by size-exclusion (HiLoad 16/600 Superdex 200 pg column; Cytiva) and anion exchange (HiTrapTM Q HP column; Cytiva) chromatography. For tetramer formation, HLA-DQ8-peptide monomer was biotinylated using BirA ligase and complexed with either Phycoerythrin (PE) Streptavidin or Allophycocyanin (APC) Streptavidin (both from BD Biosciences).

### Surface plasmon resonance measurement and analysis

HLA-DQ8-peptides were biotinylated using *E. coli* BirA and purified on a HiTrapTM Desalting Column (Cytiva). SPR measurements were performed using a BIAcore T200 (Cytiva) at RT overnight. Approximately 3000 RUs of biotinylated HLA-DQ8 bound to CLIP (as reference), glia-α1 (as control), CpepL11C, HIPL11C and HIP were immobilised in separate flow cells of a BIAcore streptavidin sensor chip (Cytiva). Serial concentrations from 200 μM to 0 μM of soluble all TCRs were diluted in SPR buffer (20 mM Hepes pH7.4, 150 mM NaCl, and 0.005% surfactant P20) and injected over the chip at a flow rate of 10 μl/min for 60 s. The association and dissociation kinetics were determined at 25 ^o^C in SPR buffer. Two to four independent experiments with replicates were performed for each TCR. The equilibrium dissociation constant K_D_ data from the BIAcore T200 was analysed using Prism 9 (GraphPad Software).

### Crystallography

Before crystallisation trials, the leucine zippers of HLA-DQ8-HIPL11C were removed with Enterokinase (EK; New England Biolabs) in EK buffer (20 mM Tris pH 8, 50 mM NaCl, 2 mM CaCl_2_). The cleaved HLA-DQ8-HIPL11C were purified via anion exchange chromatography using a HiTrapTM Q HP column (Cytiva). Crystallisation was conducted using the hanging-drop vapour-diffusion method at 20 ^o^C. HLA-DQ8-HIPL11C was complexed with TCR A1.9 or A3.10 or A2.13 at a concentration of 7 mg/ml in 10 mM Tris pH 8, 150 mM NaCl at 20 ^o^C for 5 h. Crystals of TCR-HLA-DQ8-HIPL11C complex were obtained by mixing protein samples 1:1 with reservoir solution and equilibrating with 0.5 ml reservoir solution. The TCR A3.10-HLA-DQ8-HIPL11C complex crystallised in 0.2 M potassium dihydrogen phosphate, 13% PEG 8000 with additive (10 mM calcium acetate, 10 mM sodium cacodylate pH 5.5). The TCR A1.9-HLA-DQ8-HIPL11C complex crystallised in a condition of 0.1 M calcium acetate, 0.1 M sodium cacodylate pH 5.5, 13% polyethylene glycol (PEG) 20,000. Similarly, A2.13-HLA-DQ8-HIPL11C was crystallised in 0.2 M potassium dihydrogen phosphate, 13% PEG 8000 with seeding from A1.9-HLA-DQ8-HIPL11C crystals. Crystals were cryoprotected in 20%–30% glycerol and flash-frozen in liquid N_2_ prior to data collection. Diffraction datasets were collected at the Australian Synchrotron’s MX2 beamline using an Eiger x16M detector. Data processing was performed by the molecule replacement method and processed using XDS and Aimless of the CCP4 package (*18*). Model building, refinement and validation were performed using the Coot^48^ and Phenix^49^ software packages. After completion of the structure refinement, the structures were validated using the PDB validation server. After the validation statistics confirmed the structures were renumbered according to the IMGT unique numbering system^50^. All crystallographic figures were generated using *PyMol* V2.3.2. Contact in CCP4-7.0 was used to identify interactions between residues at the interface. Bond lengths ranged from 3.5 Å or less between electron donors and acceptors (O or N) were considered hydrogen bonds (H-bonds), a salt-bridge bond was considered between negatively charged and positively charged amino acid side-chain moieties within 4.0 Å or less and van der Waals (vdW) interactions from 3.5 Å to 4 Å (20).

### HLA-DQ8-peptide circular dichroism^2^ analysis

0.2 mg/ml of HLA-DQ8-HIPL11C, -HIP or 0.3 mg/ml for HLA-DQ8-glia-α1 (control) were exchanged into CD buffer (20 mM Na_2_HPO4, 10 mM NaCl). The proteins were analysed using a Jacco815-CD spectrophotometer with a 1 mm path length cuvette at 20 ^o^C. The data of the basic spectrum of the HLA-DQ8-peptide was evaluated using C_D_ Analysis & Plotting Tool (https://capito.uni-jena.de).

### Statistics

Tabulated data were analysed in Graphpad Prism 9 (Graphpad Software). Each data set was assessed for normality using Shapiro-Wilk normality test. Differences between columns were analysed by two-tailed Student’s *t*-tests for normally distributed data. Differences between groups were analysed using one-way ANOVA with Dunnett’s post tests for normally distributed data.

### Reporting summary

Further information on research design is available in the Nature Research Reporting Summary linked to this article.

## Supplementary information

Supplementary Information

Description of Additional Supplementary Files

Supplementary Data 1

Reporting Summary

## Data Availability

The mass spectrometry proteomics data generated in this study have been deposited in ProteomeXchange Consortium via the PRIDE^51^ (https://www.ebi.ac.uk/pride/) partner repository with the dataset identifier PXD019466 and in Supplementary Data 1. The structures and structure factors for the complexes of HLA-DQ8-L11C with TCRs A2.13, A1.9 and A3.10 generated in this study have been deposited in the Worldwide Protein Data Bank (wwPDB) under accession codes PDB 6XCP (PDB 10.2210/pdb6XCP/pdb), 6XCO (PDB 10.2210/pdb6XCO/pdb) and 6XC9 (PDB 10.2210/pdb6XC9/pdb), respectively. Source data are provided as a Source Data file. Source data are provided with this paper.

## Source data

Source Data
