# Supplementary material for: T cell receptor recognition of hybrid insulin peptides bound to HLA-DQ8
Source: Nat Commun. 2021 Aug 25;12:5110. doi: 10.1038/s41467-021-25404-x (PMC8387461; doi:10.1038/s41467-021-25404-x)
Supplement: Supplementary file 1 — Supplementary Information [file 41467_2021_25404_MOESM1_ESM.pdf]

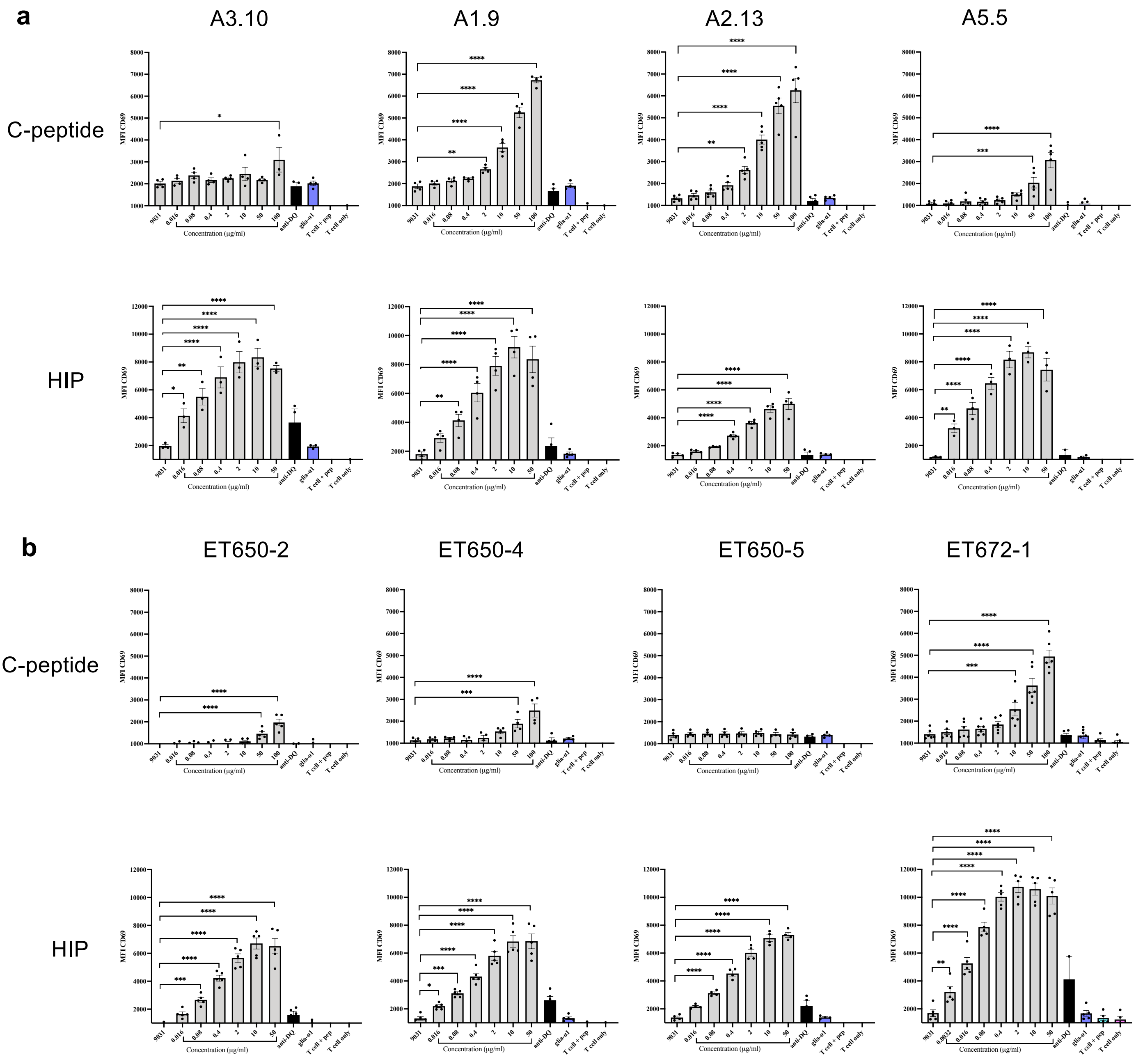
a
A3.10
A1.9
A2.13
A5.5
C-peptide
HIP
MFI CD69
Concentration (μg/ml)
9031
0.016
0.08
0.4
2
10
50
100
anti-DQ
glia-α1
T cell + pep
T cell only
b
ET650-2
ET650-4
ET650-5
ET672-1
C-peptide
HIP
0.0032

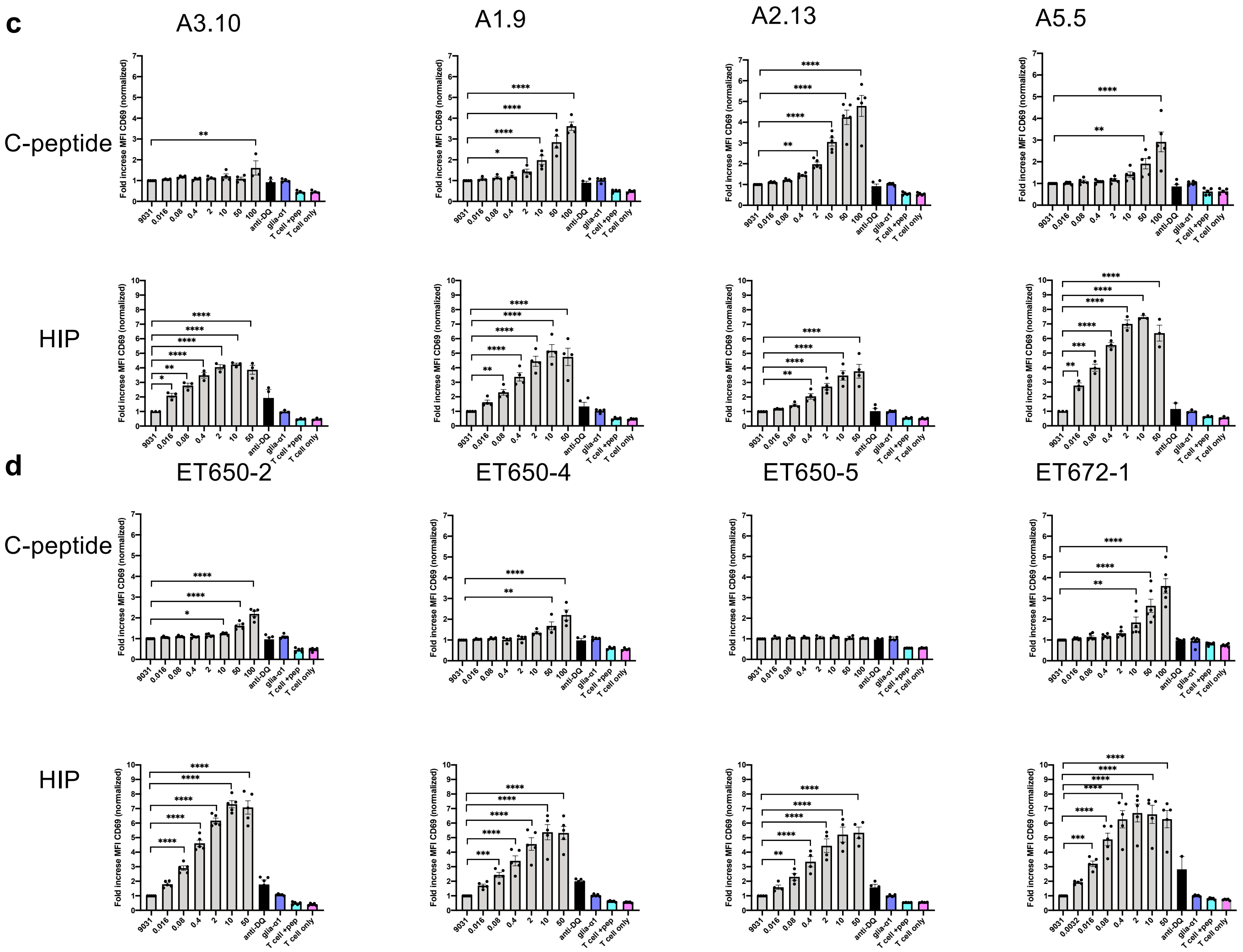
c
A3.10
A1.9
A2.13
A5.5
C-peptide
HIP
d
ET650-2
ET650-4
ET650-5
ET672-1
C-peptide
HIP
Fold increse MFI CD69 (normalized)
9031
0.0032
0.016
0.08
0.4
2
10
50
100
anti-DQ
glia-α1
T cell +pep
T cell only

e

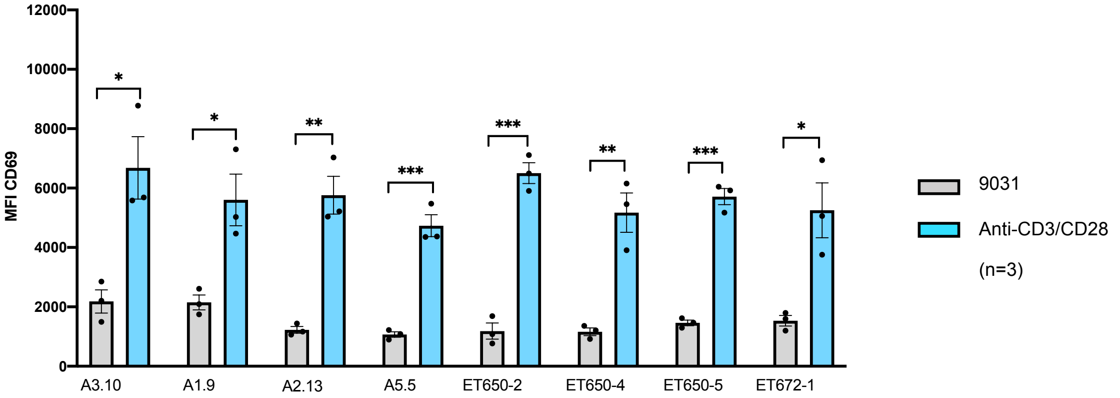

MFI CD69
12000
10000
8000
6000
4000
2000
0
*
*
**
***
***
**
***
*
A3.10
A1.9
A2.13
A5.5
ET650-2
ET650-4
ET650-5
ET672-1
9031
Anti-CD3/CD28
(n=3)

f

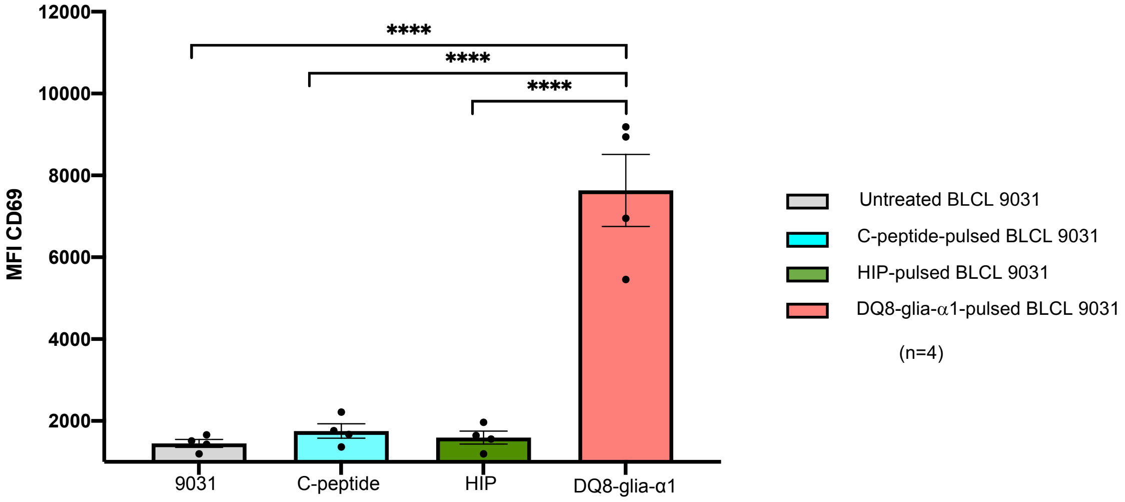

MFI CD69
12000
10000
8000
6000
4000
2000
****
****
****
9031
C-peptide
HIP
DQ8-glia-α1
Untreated BLCL 9031
C-peptide-pulsed BLCL 9031
HIP-pulsed BLCL 9031
DQ8-glia-α1-pulsed BLCL 9031
(n=4)

Supplementary Figure 1. Antigen reactivity of T cells transduced with TCRs isolated from T1D patient clones and recent onset T1D patient PBMCs. a, **b** Expression of CD69 on the surface of TCR transduced SKW3 cell lines stimulated overnight with C-peptide-pulsed BLCL 9031 (top panel), or HIP-pulsed BLCL 9031 (bottom panel). The SKW3 cell lines transduced with **a** TCRs isolated from primary T1D T clones and **b** TCRs isolated from recent onset T1D patient PBMCs (TCR name indicated above the graphs), were cultured with BLCL 9031 (no peptide) or with serial diluted concentration of peptide as indicated (grey), or 50 µg/ml C-peptide/HIP-pulsed BLCL 9031 blocked by anti-HLA-DQ mAb (clone SPV-L3; 20 µg/ml) (black), or 50 µg/ml glia-α1-pulsed BLCL 9031 (purple; control), or 50 µg/ml C-peptide/HIP peptide only (cyan; control), or T cell lines alone (pink; control). Due to the strong reactivity of the SKW-TCR E672-1 cell line to HIP, only 2 µg of HIP was used in anti-HLA-DQ mAb blocking experiments. All samples were performed in duplicates and the black dots are presented as the mean fluorescence intensity (MFI) of average of duplicated values from three to six (n=3, n=4, n=5 or n=6) independent experiments. **c** and **d** is the data presented in **a** and **b** displayed as the fold increase in CD69 expression. The fold increase in MFI CD69 expression was calculated by dividing the average MFI CD69 from each sample by the average MFI CD69 from BLCL 9031 (no peptide). The black dots are presented as fold increase in MFI CD69 from three to six (n=3, n=4, n=5 or n=6) independent experiments. **e** Expression of CD69 on the surface of T1D TCR transduced SKW3 cell lines stimulated overnight with BLCL 9031 (no peptide; grey), or anti-CD3/CD28 mAb Dynabeads (blue). The black dots represent the MFI of averaged duplicate values from three (n=3) independent experiments. **f** Expression of CD69 on the surface of HLA-DQ8-glia-α1 restricted TCR transduced SKW3.SP3.4 T cells (control). The SKW3.SP3.4 T cells were cultured overnight with BLCL 9031 (no peptide, grey), 50 µg/ml C-peptide-pulsed BLCL 9031 (cyan), 50 µg/ml HIP-pulsed BLCL 9031 (green), and 50 µg/ml DQ8-glia-α1-pulsed BLCL 9031 (orange). The black dots represent the MFI of average of duplicated values from four (n=4) independent experiments. For each of the analyses, the MFI CD69 of each sample was compared to the MFI CD69 of BLCL 9031 (no peptide) or of C-peptide-pulsed BLCL 9031, or HIP-pulsed BLCL 9031. For **a** to **f** *$P < 0.05$, **$P < 0.01$, ***$P < 0.002$, ****$P < 0.0001$ and error bars represent ± SEM. P values were determined by one-way ANOVA with Dunnett's multiple comparison testing (**a** to **d**) and two-tailed Student's t-test (**e** and **f**). Source data are provided as a Source Data file including the exact *P* values for **a** to **f**.

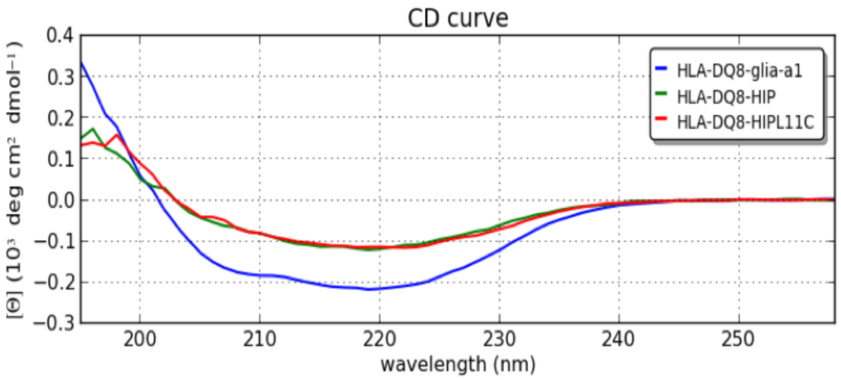

Supplementary Figure 2. Analysis of HLA-DQ8-HIP and HLA-DQ8-HIPL11C secondary structures. Circular dichroism [2] spectra of HLA-DQ8-HIP, HLA-DQ8-HIPL11C and HLA-DQ8-glia-α1 (control) are shown as green, red and blue curves, respectively. Their measured molar ellipticity was plotted against wavelength from 200 nm - 250 nm. Source data are provided as a Source Data file.

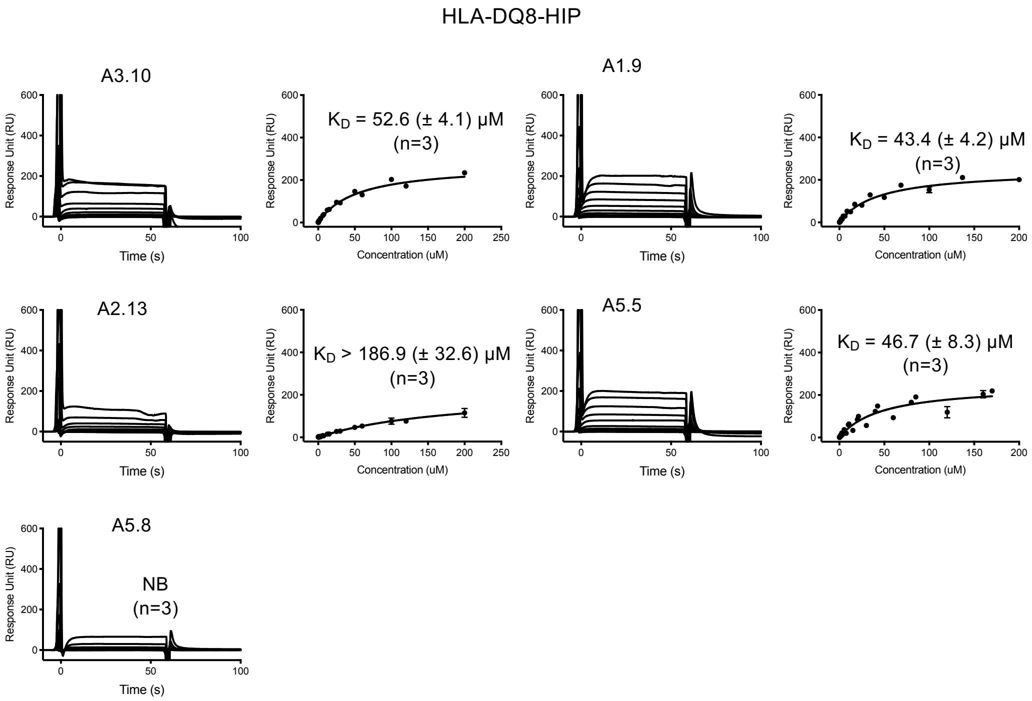

Supplementary Figure 3. SPR binding affinity measurements of TCRs for HLA-DQ8-HIP native (P11 Leu) complex. Approximately 3000 RUs of the biotinylated HLA-DQ8-HIP native (P11 Leu) were immobilized in separate flow cells of an SA sensor chip. Each TCR sample was analysed in three (n=3) independent experiments conducted. A serial dilution of TCRs between concentrations of 200 μM to 0 μM (TCRs A2.13, A3.10, A1.9) or 170 μM to 0 μM (TCRs A5.5 and A5.8) were injected at a flow rate of 10 μl/min for 60 s. The average of the SPR signal (RU) from different batches of HLA-DQ8-HIP complex responding to each TCR was plotted against time to visualize the binding and dissociation of the TCRs. Data are mean ± standard error of mean (SEM). N.B. = no binding of TCR. Source data are provided as a Source Data file.

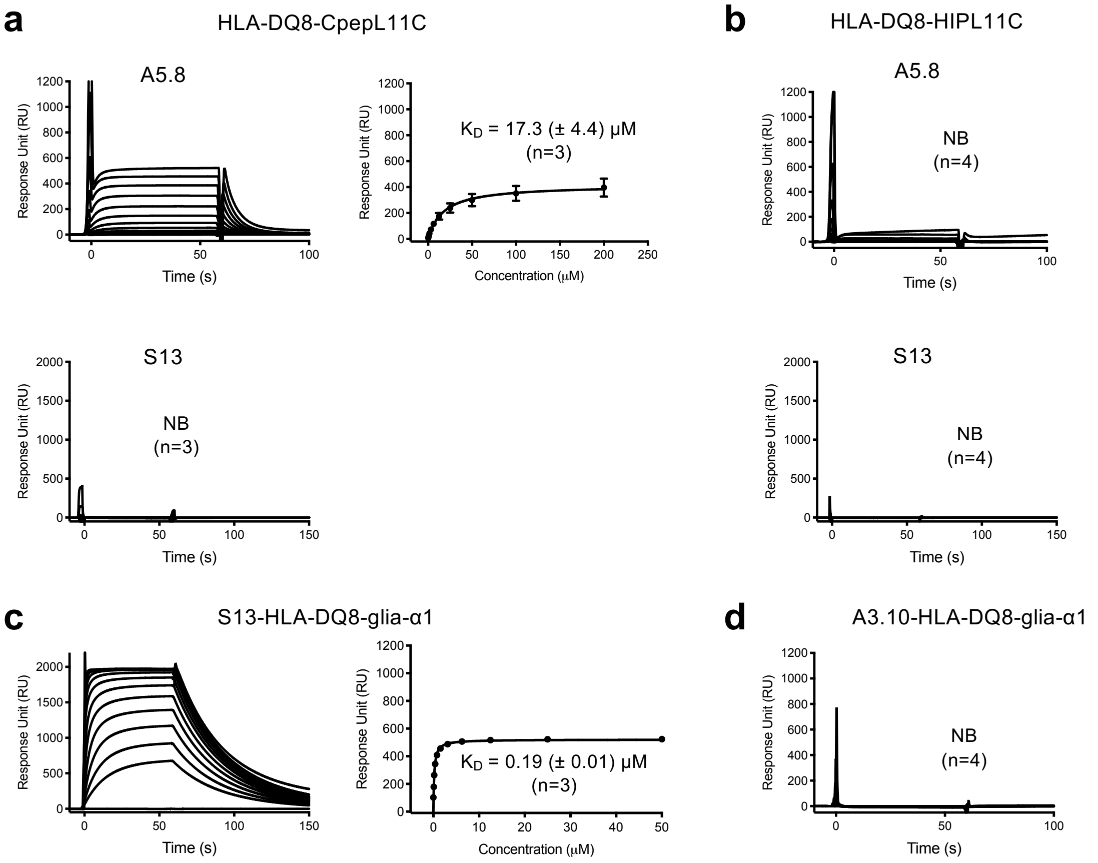

Supplementary Figure 4. SPR binding affinity measurements of TCR A5.8 with HLA-DQ8-CpepL11C, and HLA-DQ8-HIPL11C. Binding analysis of TCR A5.8 and TCR S13 (control, an HLA-DQ8-glia-α1 restricted TCR) for **a** HLA-DQ8-CpepL11C and **b** HLA-DQ8-HIPL11C was determined using SPR. A concentration series from 160 μM to 0 μM of TCR A5.8 or 100 μM to 0 μM of TCR S13 was passed over surface immobilized a HLA-DQ8-CpepL11C and b HLA-DQ8-HIPL11C. **c** The binding affinity of TCR S13 and TCR A3.10 to HLA-DQ8-glia-α1 (controls). Measured response curves of the single dilution series for each TCR shown in **a** (left column), **b**, **c** (left column) and **d**. Right columns in **a** and **c**: Curve fits for **a** TCR A5.8 - HLA-DQ8–CpepL11C and **c** TCR S13 - HLA-DQ8-glia-α1 and K_D determination using single ligand binding model. Each TCR sample was analysed in duplicate, and three (n=3) or four (n=4) (as indicated) independent experiments were conducted and measurements were combined after normalizing each equilibrium response curve against the calculated response maximum. Data are mean ± standard error of mean (SEM). N.B. = no binding of TCR. Source data are provided as a Source Data file.

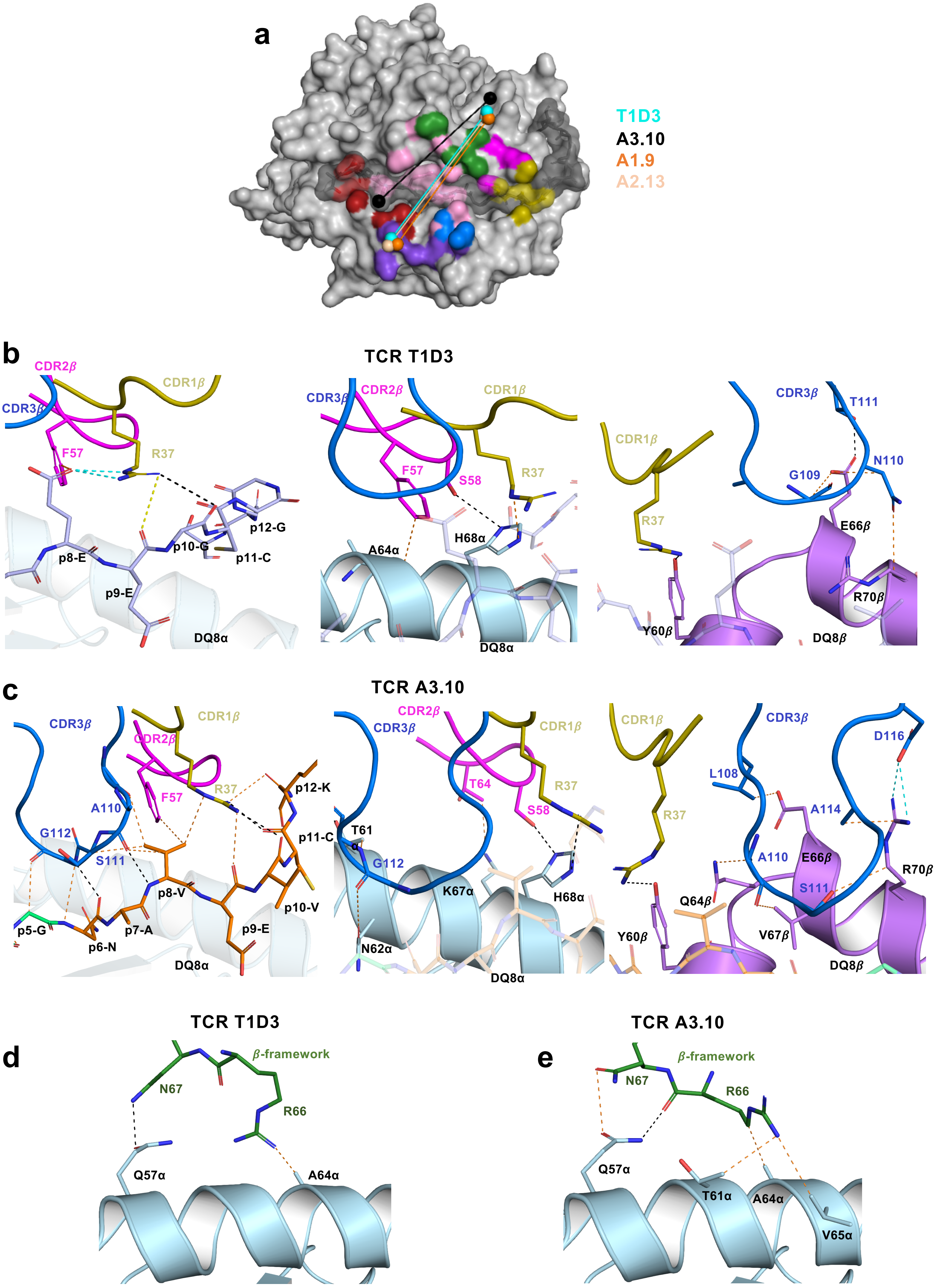

Supplementary Figure 5

Supplementary Figure 5. Comparison of T1D3-HLA-DQ8-8E9E11ss and HLA-DQ8-HIPL11C TCR ternary structures. a atomic footprint of TCR T1D3 on the surface of HLA-DQ8-8E9E11ss. HLA-DQ8-HIPL11C restricted TCRs have similar Vα- and Vβ- chain centre of mass positions (dots) and approximate docking angles: TCR T1D3 (cyan), TCR A3.10 (black), TCR A1.9 (orange), and TCR A2.13 (wheat). HLA-DQ8 α- and β-chain are coloured in light-grey, InB-8E9E11ss peptide is in dark-grey. The CDR loops 1α, 2α, 3α, α -framework, 1β, 2β, 3β, and β-framework are coloured red, purple, pink, neon-green, gold, magenta, blue, and green, respectively. TCR footprint colours are in accordance with the nearest TCR contact residues. **b** TCR T1D3 and **c** TCR A3.10, interacting with the peptide (left) HLA-D8 a-chain (middle) and b-chain (right) show similar interactions between the two TCRs. **d** and **e** Conserved TRBV5-1 β-framework interactions of TCRs T1D3 and A3.10 with HLA-DQ8 α-chain. H-bonds are denoted as black dashed lines, vdW as orange dashed lines, and salt bridges as cyan dashed lines. Contact residues were cut off at 4 . TCR T1D3 residues were numbered according to the IMGT unique numbering system.

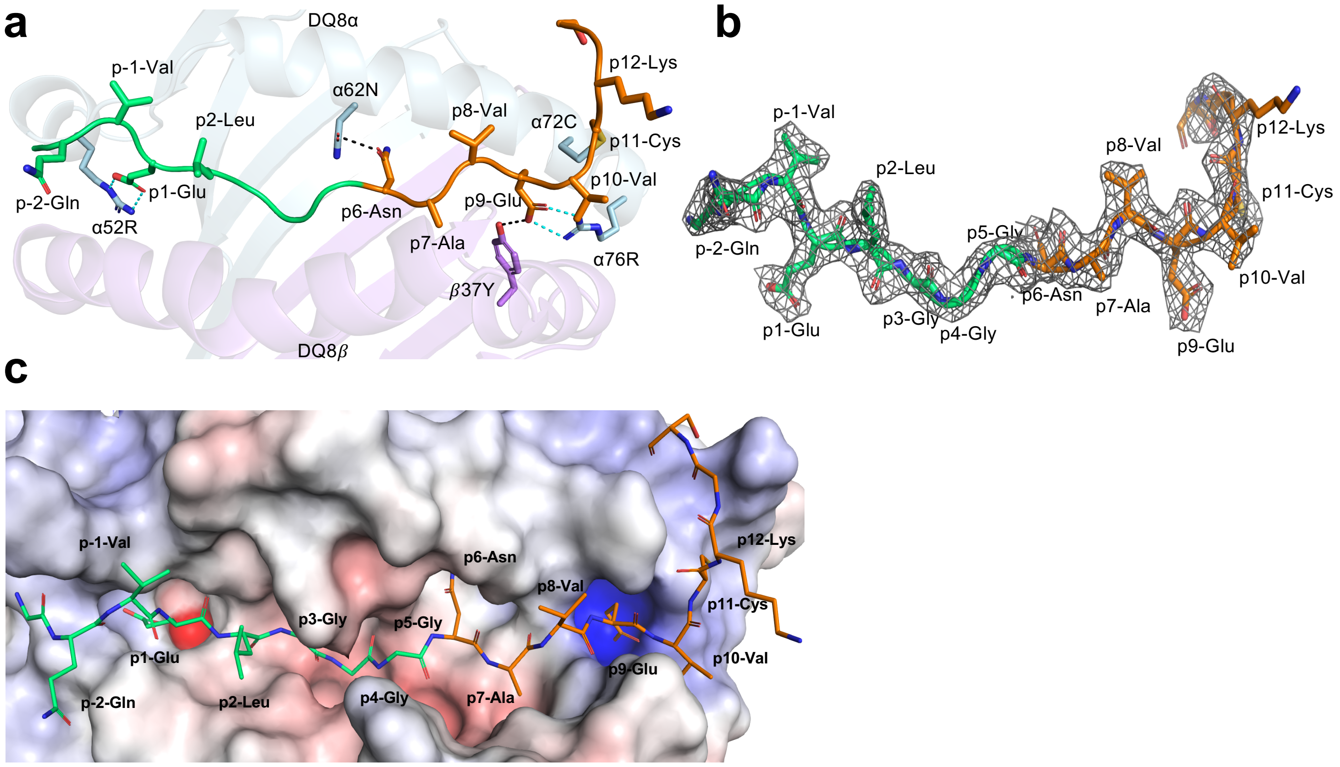

Supplementary Figure 6. HIPL11C bound to HLA-DQ8. a Top view of HIPL11C bound to HLA-DQ8. The HLA-DQ8 a-chain is colored in neon, β-chain in light cyan, HIPL11C is colored such that the C-peptide fragment is in lime-green and the IAPP2 fragment in orange. The interactions between the HIPL11C and HLA-DQ8 are shown with oxygen atoms in red and nitrogen atoms in blue. H-bonds and salt bridge are denoted as black and cyan dashed lines, respectively. **b** 2Fo-Fc electron density map of HIPL11C bound to HLA-DQ8 is shown and labelled with the position of each residue in the HLA-DQ8 binding groove. **c** The top view of HLA-DQ8 (in grey) is presented as surface bound HIPL11C (C-peptide in lime-green, IAPP2 in -orange) peptide. p1-Glu, p6-Asn, and p9-Glu buried in the HLA-DQ8 binding pockets, whilst p-1-Val, p2-Leu, and p8-Val point upward and potentially accessible to TCRs.

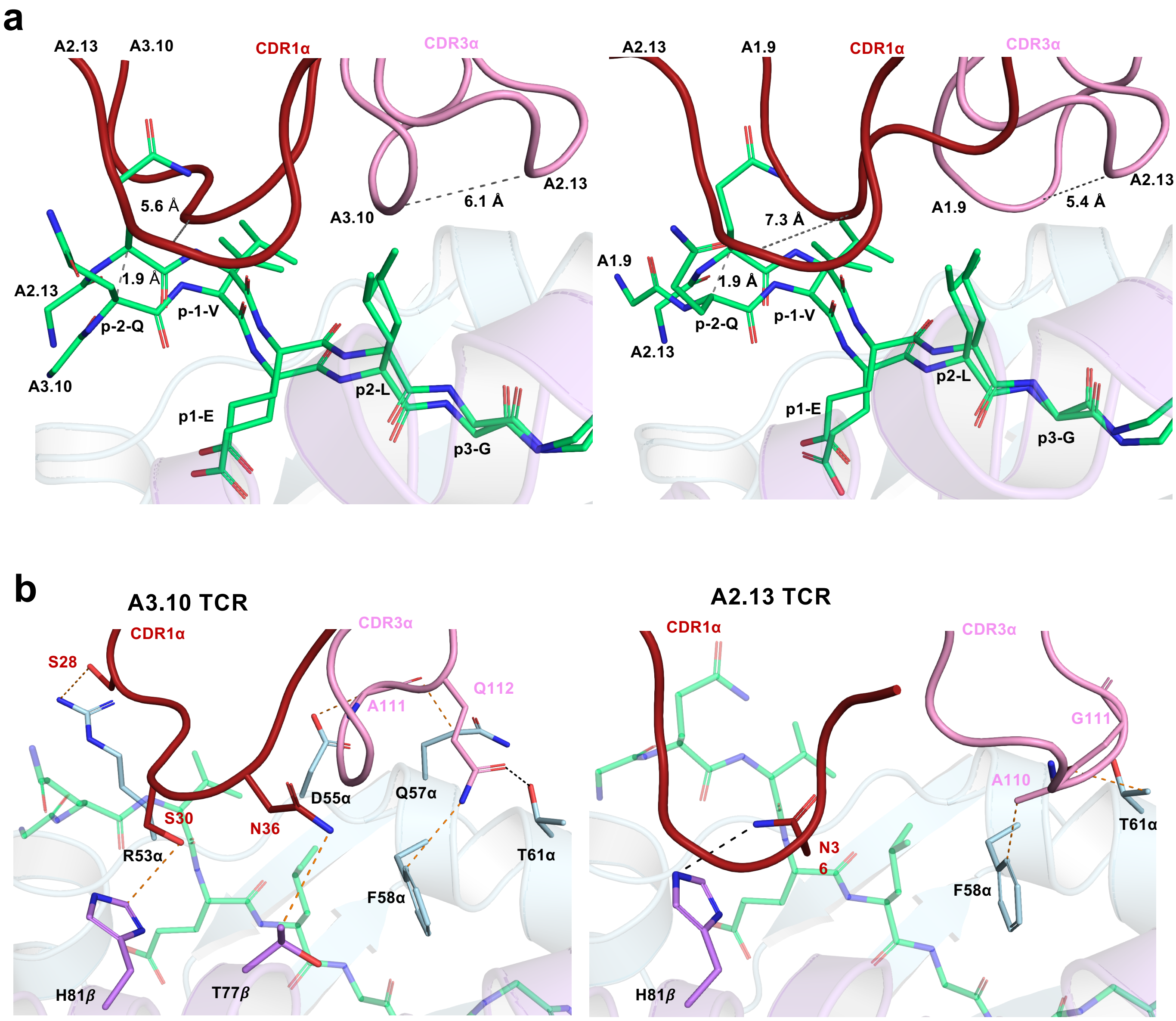

Supplementary Figure 7. Comparison of TCRs with differential preference for HIP or proinsulin C-peptide. a superposition of HLA-DQ8-HIPL11C ternary complex structures with A3.10 and A2.13 TCRs (left), A1.9 and A2.13 TCRs (right). The CDR1α and CDR3α loops are colored in red and pink, respectively. The HLA-DQ8 α-chain and β-chain are colored in neon and in light cyan, respectively. Portion of HIP derived from C-peptide is colored in lime-green. **b** Conserved interactions between CDR1α and CDR3α of TCR A3.10 (left) and TCR A2.13 (right) with HLA-DQ8 His81β Phe58α, Thr61α. Black dashed lines, H-bond; orange dashed lines; vdW; gray dashed lines, distance measured in .

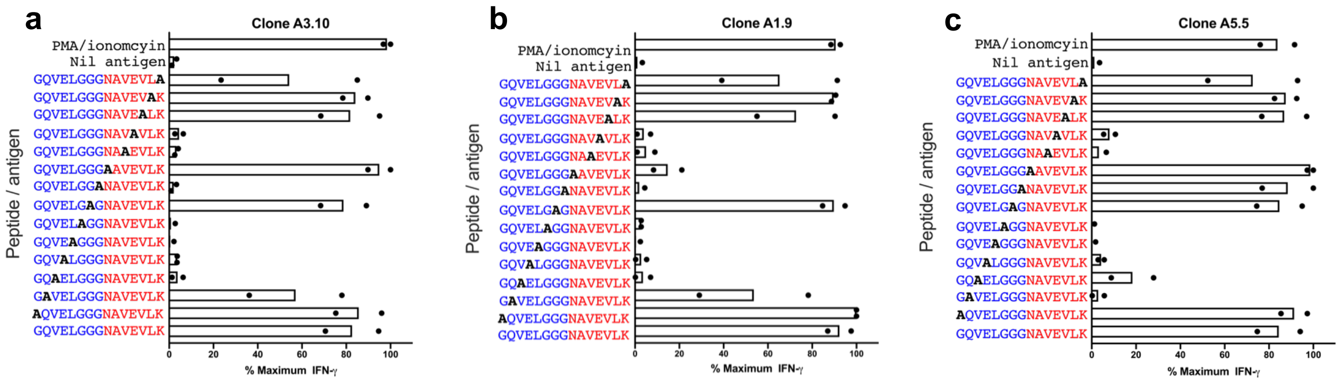

Supplementary Figure 8. Fine specificity toward HIP determinant. The sensitivity of three HIP specific CD4^{+} T cell clones, A3.10, A1.9 and A5.5 was analysed using a panel of alanine substituted peptides. The amino acids from C-peptide are in blue text and the IAPP2 derived amino acids are in red. The reactivity pattern of HIP restricted T cell clones A3.10 **a**, A1.9 **b** and A5.5 **c** to HIP with single alanine substitutions (black type) at each position P-3 to P12 are shown. IFNγ was measured in triplicate by ELISA, concentrations are shown in pg/ml. Each point represents the IFNγ concentration from one of two experiments.

ET650

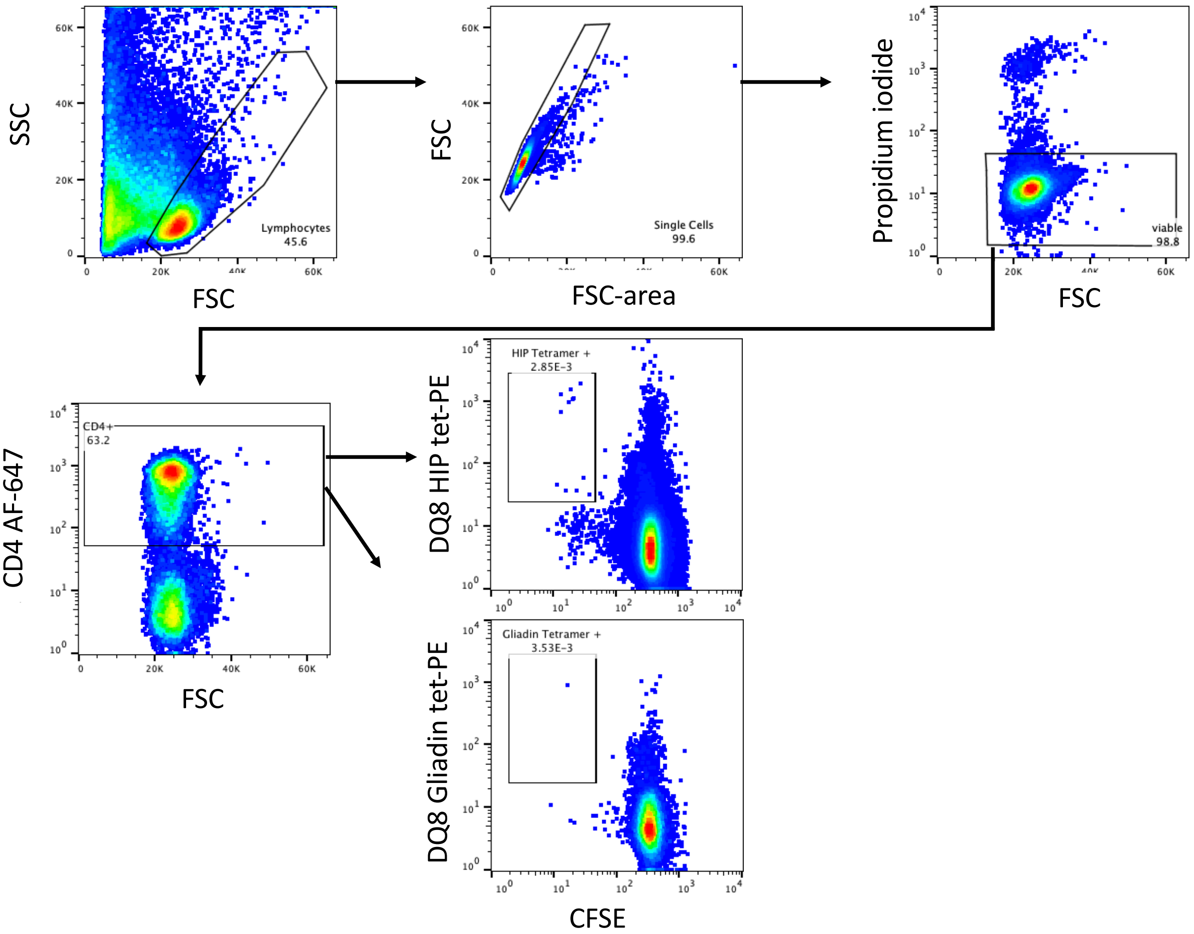

Supplementary Figure 9. CFSE proliferation assay-tetramer FACS. Representative FACS plot showing staining of PBMC labelled with CFSE and cultured with HIP peptide (**patient ET650**). After gating on FSC/SSC and PI^{-} cells, CD4^{+} cells were gated (left hand plot), from the CD4^{+} cells the CFSE vs tetramer staining was determined (Right hand plot). The number of CFSE^{dim}, HIP responsive cells, that stained with HLA-DQ8-HIPL11C or HLA-DQ8-glia-α1 (negative control) tetramer are shown.

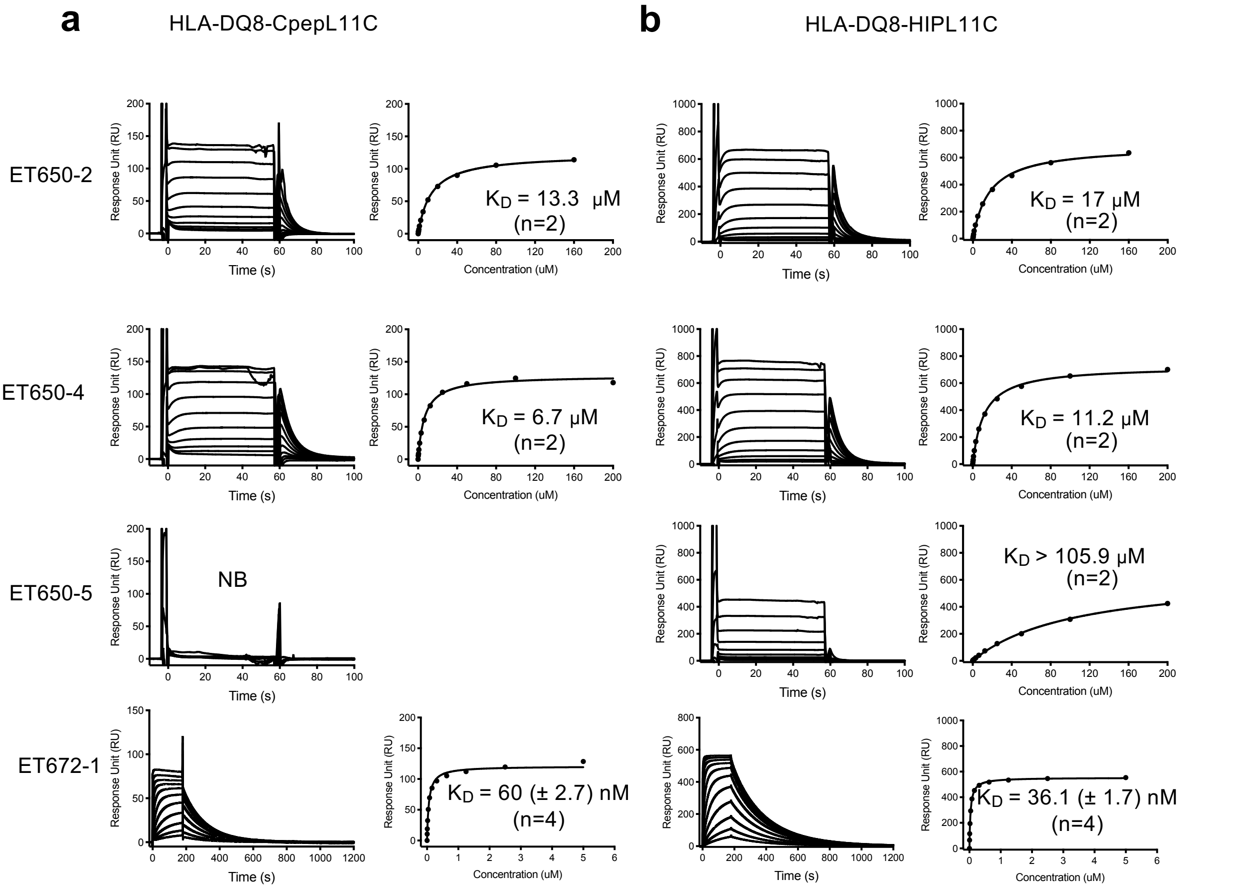

Supplementary Figure 10. Affinity determination for the HLA-DQ8-HIPL11C responsive TCRs. The affinity of TCR ET650-2, ET650-4, ET650-5, and ET672-1 for **a** HLA-DQ8-CpepL11C and **b** HLA-DQ8-HIPL11C was determined using SPR. A concentration series from 200 μM or 5 μM to 0 μM of TCRs was passed over surface of a SA chip with immobilized HLA-DQ8-CpepL11C and HLA-DQ8-HIPL11C. Left columns in **a** and **b** are measured response curves of the single dilution series for each TCR. Right columns in **a** and **b** are curve fits for TCR-HLA-DQ8-CpepL11C, and HLA-DQ8-HIPL11C and K_D determined from two (n=2) or four (n=4) independent experiments as indicated, and sample analysed in duplicate using different SA Chips. NB, no-binding. Data are mean ± standard error of mean (SEM). Source data are provided as a Source Data file.

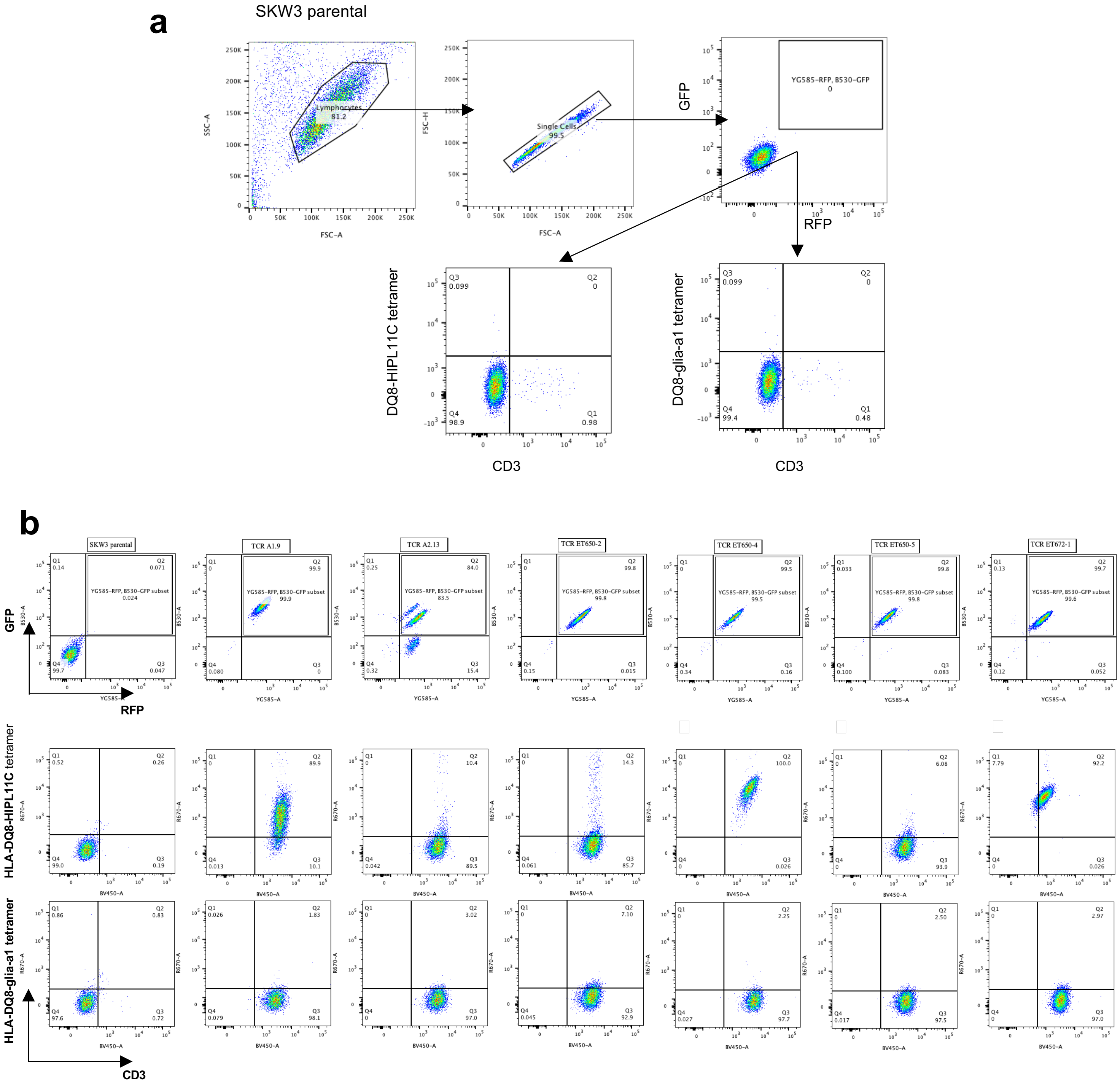

Supplementary Figure 11. Tetramer staining of T cells transduced with TCRs isolated from recent onset T1D patient PBMCs. a the gating strategies of SKW3 parental cell (control). Firstly, the lymphocyte population was selected and then single cells. Secondly, double-positive GFP (TCR a-chain reporter) and RFP (TCR b-chain reporter) cells were selected from the single-cell population followed by selection of double-positive CD3 and HLA-DQ8-HIPL11C/HLA-DQ8-glia-a1 tetramer. **b** Top panel, flow cytometry plots of double positive GFP (a-chain) and RFP (b-chain) population of: SKW3 parental (negative control), SKW3 TCR A1.9, A2.13, ET650-2, ET650-4, ET650-5, and ET672-1 transduced cell lines. Lower panels, flow cytometry plots depicting the number of tetramer positive SKW3 parental (negative control), SKW3 TCRs A1.9, A21.13, ET650-2, ET650-4, ET650-5, and ET672-1 transduced cell lines co-stained with HLA-DQ8-HIPL11C-APC tetramer and CD3-V450 (middle panel) or HLA-DQ8-glia-a1-APC tetramer and CD3-V450 (bottom panel).

Supplementary Table 1 TCR Gene Usage of HLA-DQ8-C-peptide-responsive TCRs

Clone	TRAV	TRAJ	CDR1α	CDR2α	CDR3α	TRBV	TRBJ	TRBD	CDR1β	CDR2β	CDR3β
A3.10	38-1*03	54*01	TSESNYY	QEAYKQQ	CAFFGQGAQKLVF	5-1*01	2-3*01	2*01	SGHRS	YFSETQ	CASSLSASGGATDTQYF
A1.9	20*02	7*01	VSGLRG	LYSAGE	CAVQAGGNNRLAF	5-1*01	1-2*01	1*01	SGHRS	YFSETQ	CASSLERDGYTF
A2.13	26-1*01	39*01	TISGNEY	GLKN	CIVSHNAGNMLTF	5-1*01	2-5*01	2*01	SGHRS	YFSETQ	CASSLERETQYF
A5.5	26-1*01	54*01	TISGNEY	GLKN	CIVRVEIQGAQKLVF	5-1*01	2-5*01	1*01	SGHRS	YFSETQ	CASSLGPGQRETQYF
A5.8	26-1*01	21*01	TISGNEY	GLKN	CIAIYNFNKFYF	5-1*01	1-6*02	2*01	SGHRS	YFSETQ	CASSLEASSYNSPLHF

(Adapted from: Pathiraja V, et al. Proinsulin-specific, HLA-DQ8, and HLA-DQ8-transdimer-restricted CD4+ T cells infiltrate islets in type 1 diabetes. Diabetes 64, 172-182 (2015).)

Supplementary Table 2 The Equilibrium Constant (K_D) of Human TCRs toward pHLA-DQ8

TCR	HLA-DQ8-CpepL11C	HLA-DQ8-HIPL11C	HLA-DQ8-HIP
**A3.10**	> 296.2 ± 112.3 μM (n=3)	52.5 ± 2.2 μM (n=4)	52.6 ± 4.1 μM (n=3)
**A1.9**	11.8 ± 3.1 μM (n=3)	35.5 ± 1.5 μM (n=4)	43.4 ± 4.2 μM (n=3)
**A2.13**	6.6 ± 1.7 μM (n=3)	92.7 ± 3.3 μM (n=4)	>186.9 ± 32.6 μM (n=3)
**A5.5**	21.5 ± 5.0 μM (n=3)	39.4 ± 2.1 μM (n=4)	46.7 ± 8.3 μM (n=3)
**A5.8**	17.3 ± 4.4 μM (n=3)	NB (n=4)	NB (n=3)
**S13***	NB (n=2)	NB (n=2)	NB (n=2)

* -ve control: S13 TCR is an HLA-DQ8-glia-α1-restricted TCR [26]

Supplementary Table 3 Data Collection and Refinement Statistics

	A3.10-HLA-DQ8-HIPL11C	A1.9-HLA-DQ8-HIPL11C	A2.13-HLA-DQ8-HIPL11C
**Wavelength (Å)**	0.9537	0.9537	0.9572
**Space group**	P 1 21 1	P 1 21 1	P 21 21 21
**Cell dimensions (Å) (a, b, c, α, β, γ)**	65.993 114.79 153.926 90 90.066 90	69.29 104.31 69.32 90.00 114.91 90.00	65.977 116.788 124.187 90 90 90
**Resolution (Å)**	46.01 - 2.4 (2.486 - 2.4)	40.14 – 2.90 (3.08 - 2.90)	42.54 - 3.3 (3.418 - 3.3)
**Total reflections**	178420 (17736)	39659 (3958)	30009 (2932)
**Unique reflections**	89707 (8924)	19,889 (1,983)	15005 (1466)
**Multiplicity**	2.0 (2.0)	2.0 (2.0)	2.0 (2.0)
**Completeness (%)**	99.94 (99.98)	99.94 (99.95)	99.87 (100.00)
**Mean I/σ(I)**	8.82 (2.13)	5.7 (1.94)	8.46 (1.58)
**Wilson B factor**	38.16	49.79	79.38
**R_{merge}**	0.05908 (0.4443)	0.0853 (0.4017)	0.0686 (0.4183)
**R_{meas}**	0.08355 (0.6283)	0.1206 (0.5681)	0.09701 (0.5916)
**CC1/2**	0.996 (0.667)	0.985 (0.724)	0.994 (0.763)
**R_{work} (%)**	19.12 (25.66)	19.97 (28.25)	24.08 (30.91)
**R_{free} (%)**	22.44 (31.2)	24.20 (34.27)	26.84 (30.79)
**Number of non-hydrogen atoms**	13,675	6,447	6,359
**macromolecules**	13,062	6,297	6,285
**ligands**	222	83	39
**solvent**	391	67	35
**Protein residues**	1,656	802	809
**RMS (bonds)**	0.004	0.004	0.002
**RMS (angles)**	0.64	0.56	0.53
**Ramachandran favored (%)**	97.98	95.05	92.96
**Ramachandran allowed (%)**	2.02	4.7	6.67
**Ramachandran outliers (%)**	0.00	0.25	0.38
**Rotamer outliers (%)**	1.05	1.90	1.92
**Clashscore**	3.10	5.48	5.49
**Average B factor**	49.90	56.9	77.81
**macromolecules**	48.95	56.43	77.73
**ligands**	112.08	105.13	113.33
**solvent**	46.34	41.90	53.17

Supplementary Table 4 Contacts at the human TCR A3.10-HLA-DQ8-HIPL11C interface. Contact measured with a 4 Å interatomic cutoff as described in Methods

TCR CDR Loop	Residue	Atom	pHLA-II chain	Residue	Atom	Interaction type
CDR1α	Ser28		A	Arg53		VDW
	Ser28		P	Gln-2		VDW
	Ser28	O	P	Val-1	N	H-bond, VDW
	Glu29		P	Val-1, Gln-2		VDW
	Ser30		P	Gln-2		VDW
	Ser30	Oγ	P	Val-1	O	H-bond, VDW
	Ser30		B	His81		VDW
	Asn36		P	Leu2		VDW
	Asn36		B	Thr77		VDW
	Tyr38	Oη	B	Arg70	Nη2	H-bond, VDW
CDR2α	Tyr59		B	Thr77, His81		VDW
CDR3α	Gln109	Oε1	B	Arg70	Nη2	H-bond
	Gln109		A	Phe58		VDW
	Gln109		P	Leu2		VDW
	Gly110		P	Leu2		VDW
	Gly110		A	Asp55, Gln57		VDW
	Ala111		A	Asp55, Gln57		VDW
	Gln112		A	Phe58		VDW
	Gln112	Oε1	A	Thr61	Oγ1	H-bond
CDR1β	Arg37		A	His68		VDW
	Arg37	Nη2	B	Tyr60	Oη	H-bond
	Arg37		P	Val8, Val10, Lys12		VDW
	Arg37	Nη2	P	Glu9	O	H-bond
CDR2β	Phe57		P	Val8		VDW
	Ser58	Oγ	A	His68	Nδ1	H-bond
	Thr64		A	Lys67		VDW
FWβ	Arg66		A	Ala64, Val65, Thr61		VDW
	Arg66	O	A	Gln57	Nε2	H-bond, VDW
	Asn67		A	Gln57		VDW
CDR3β	Leu108		B	Glu66		VDW
	Ser109		P	Val8		VDW
	Ala110	O	P	Val8	N	H-bond, VDW
	Ala110		P	Ala7		VDW
	Ala110		B	Gln64, Val67		VDW
	Ser111		B	Arg70		VDW
	Ser111		P	Ans6, Ala7, Val8		VDW
	Gly112	N	P	Asn6	O	H-bond
	Gly112		P	Gly5		VDW
	Gly112		A	Thr61, Asn62		VDW
	Ala114		B	Arg70		VDW
	Asp116	Oδ2	B	Arg70	Nη1	Salt-bridge, VDW

Supplementary Table 5 Contacts at the human TCR A1.9-HLA-DQ8-HIPL11C interface. Contact measured with a 4Å interatomic cut off as described in Methods

TCR CDR Loop	Residue	Atom	pHLA-II chain	Residue	Atom	Interaction type
CDR1α	Ser28		A	Arg53		VDW
	Ser28	Oγ	P	Val -1	N	H-bond, VDW
	Ser28		P	Gln-2		VDW
	Gly29	N	P	Gln-2	Oε1	H-bond
	Gly29		P	Val-1, Leu2		VDW
	Leu36		P	Leu2		VDW
	Arg37		P	Leu2		VDW
CDR2α	Tyr57		B	Glu66, Glu69, Ala73, Arg70		VDW
	Ser58		B	Asp76		VDW
FWα	Lys66	N	B	Glu69	Oε1	Salt-bridges
CDR3α	Gly109		A	Asp55, Phe58		VDW
	Gly109		P	Leu2		VDW
	Gly110	N	A	Asp55	Oδ2	H-bond, VDW
	Gly110		A	Phe58, Gln57		VDW
	Gly110	O	A	Thr61	Oγ1	H-bond
	Asn111		A	Asp55, Gln57		VDW
	Asn112		A	Thr61		VDW
CDR1β	Gly28		P	Lys12		VDW
	Arg37		P	Val8, Val10, Lys12		VDW
	Arg37	Nη2	P	Cys11	O	H-bond
	Arg37	Nη2	P	Glu9	O	H-bond, VDW
	Arg37	Nη1	B	Tyr60	OH	H-bond, VDW
	Arg37		A	His68		VDW
CDR2β	Phe57		A	Ala64		VDW
	Phe57		P	Val8		VDW
FWβ	Arg66	O	A	Gln57	Nε2	H-bond, VDW
	Arg66		A	Thr61, Ala64, Val65		VDW
	Asn67	Nδ2	A	Gln57	Nε2	H-bond, VDW
	Asn67	Nδ2	A	Thr61	Oγ1	H-bond, VDW
CDR3β	Leu108		B	Tyr60, Gln64, Val67		VDW
	Glu109		P	Asn6		VDW
	Arg110		P	Gly5, Asn6		VDW
	Arg110		A	Phe58, Thr61, Val65		VDW
	Arg110	Nη2	A	Asn62	Oδ1	H-bond, VDW
	Asp111		B	Glu66, Val67		VDW
	Asp111	Oδ1	B	Arg70	Nε	Salt-bridge

Supplementary Table 6 Contacts at the human TCR A2.13-HLA-DQ8-HIPL11C interface. Contact measured with a 4 Å interatomic cutoff as described in Methods

TCR CDR Loop	Residue	Atom	pHLA-II chain	Residue	Atom	Interaction type
CDR1α	Ser29		P	Gln-2		VDW
	Gly30		B	Asp76, His81		VDW
	Asn36		B	Thr77		VDW
	Asn36	Nδ2	B	His81	Nε2	H-bond, VDW
	Asn36		P	Val-1, Leu2		VDW
	Glu37	Oε2	P	Gln-2	Nε2	H-bond
	Tyr38		B	Arg70, Ala73, Thr77		VDW
FWα	His55		B	Glu69		VDW
CDR2α	Leu57		B	Thr77		VDW
CDR3α	His108		P	Gln-2, Leu2		VDW
	Ala110		P	Leu2		VDW
	Ala110		A	Phe58, Thr61		VDW
	Gly111		A	Thr61		VDW
CDR1β	Arg37	Nη1	A	His68	Nε2	H-bond
	Arg37		P	Val8, Glu9, Cys11		VDW
	Arg37	Nη2	P	Cys11	O	H-bond
CDR2β	Phe57		P	Val8		VDW
	Phe57		A	Ala64		VDW
FWβ	Arg66	O	A	Gln57	Nε2	H-bond, VDW
	Arg66		A	Ala64, Val65		VDW
	Arg66	Nη1	A	Thr61	O	H-bond, VDW
	Asn67		A	Gln57		VDW
CDR3β	Leu108		B	Gln64, Glu66, Val67		VDW
	Glu109	Oε2	P	Asn6	O	H-bond
	Glu109		P	Val8		VDW
	Arg110		A	Phe58, Thr61, Asn62		VDW
	Arg110		B	Arg70		VDW
	Arg110		P	Gly5		VDW
	Arg110	Nη1	P	Asn6	N	H-bond
	Glu111		B	Glu66		VDW
	Glu111	Oε1	B	Arg70	Nη2	Salt bridge
	Tyr114	OH	B	Glu66	Oε2	H-bond, VDW

Supplementary Table 7 T1D Patient HLA typing

Patient	HLA-DRB1*	DQA1*	DQB1*	DPA1*	DPB1*
ET600	04:01;-	**03:01;** -	**03:02**; -	ND	ND
ET651	04:04; 07:01	02:01; **03:01**	02:02; **03:02**	01:03	04:02
ET672	03:01; 04:01:03	03:03; 05:01	02:01; 03:02	01:03	04:01

Supplementary Table 8 Primers used for single cell PCR amplification of *TRAV/TRBV* gene segments

Gene primer	External primer sequence 5' - 3'	Internal primer sequence 5' - 3'
TRAV1	AACTGCACGTACCAGACATC	GCACCCACATTTCTKTCTTAC
TRAV2	GATGTGCACCAAGACTCC	CACTCTGTGTCCAATGCTTAC
TRAV3	AAGATCAGGTCAACGTTGC	ATGCACCTATTCAGTCTCTGG
TRAV4	CTCCATGGACTCATATGAAGG	ATTATATCACGTGGTACCAACAG
TRAV5	CTTTTCCTGAGTGTCCGAG	TACACAGACAGCTCCTCCAC
TRAV6	CACCCTGACCTGCAACTATAC	TGGTACCGACAAGATCCAG
TRAV7	GCAAAATACAGGGATGGG	TATGAGAAGCAGAAAGGAAGAC
TRAV8-1	CTCACTGGAGTTGGGATG	GTCAACACCTTCAGCTTCTC
TRAV8-2, 8-4	CACTGTCTCTGAAGGAGCC	TTTGAGGCTGAATTTAAGAGG
TRAV8-3	GCCACCCTGGTTAAAGG	AGAGTGAAACCTCCTTCCAC
TRAV8-6	GAGCTGAGGTGCAACTACTC	AACCAAGGACTCCAGCTTC
TRAV8-7	CTAACAGAGGCCACCCAG	ATCAGAGGTTTTGAGGCTG
TRAV9-1, 9-2	TGGTATGTCCAATATCCTGG	GAAACCACTTCTTTCCACTTG
TRAV10	CAAGTGGAGCAGAGTCCTC	GAAAGAACTGCACTCTTCAATG
TRAV12-1, 12-2, 12-3	CARTGTTCCAGAGGGAGC	AAGATGGAAGGTTTACAGCAC
TRAV13-1	CATCCTTCAACCCTGAGTG	TCAGACAGTGCCTCAAACTAC
TRAV13-2	CAGCGCCTCAGACTACTTC	CAGTGAAACATCTCTCTCTGC
TRAV14	AAGATAACTCAAACCCAACCAG	AGGCTGTGACTCTGGACTG
TRAV16	AGTGGAGCTGAAGTGCAAC	GTCCAGTACTCCAGACAACG
TRAV17	GGAGAAGAGGATCCTCAGG	CCACCATGAACTGCAGTTAC
TRAV18	TCCAGTATCTAAACAAAGAGCC	TGACAGTTCCTTCCACCTG
TRAV19	AGGTAACTCAAGCGCAGAC	TGTGACCTTGGACTGTGTG
TRAV20	CACAGTCAGCGGTTTAAGAG	TCTGGTATAGGCAAGATCCTG
TRAV21	TTCCTGCAGCTCTGAGTG	AACTTGGTTCTCAACTGCAG
TRAV22	GTCCTCCAGACCTGATTCTC	CTGACTCTGTGAACAATTTGC
TRAV23	TGCTTATGAGAACACTGCG	TGCATTATTGATAGCCATACG
TRAV24	CTCAGTCACTGCATGTTCAG	TGCCTTACACTGGTACAGATG
TRAV25	GGACTTCACCACGTACTGC	TATAAGCAAAGGCCTGGTG
TRAV26-1	GCAAACCTGCCTTGTAATC	CGACAGATTCACTCCCAG
TRAV26-2	AGCCAAATTCAATGGAGAG	TTCACTTGCCTTGTAACCAC
TRAV27	TCAGTTTCTAAGCATCCAAGAG	CTCACTGTGTACTGCAACTCC
TRAV29	GCAAGTTAAGCAAAATTCACC	CTGCTGAAGGTCCTACATTC
TRAV30	CAACAACCAGTGCAGAGTC	AGAAGCATGGTGAAGCAC
TRAV34	AGAACTGGAGCAGAGTCCTC	ATCTCACCATAAACTGCACG
TRAV35	GGTCAACAGCTGAATCAGAG	ACCTGGCTATGGTACAAGC
TRAV36	GAAGACAAGGTGGTACAAAGC	ATCTCTGGTTGTCCACGAG
TRAV38-1, 38-2	GCACATATGACACCAGTGAG	CAGCAGGCAGATGATTCTC
TRAV39	CTGTTCCTGAGCATGCAG	TCAACCACTTCAGACAGACTG
TRAV40	GCATCTGTGACTATGAACTGC	GGAGGCGGAAATATTAAAGAC
TRAV41	AATGAAGTGGAGCAGAGTCC	TTGTTTATGCTGAGCTCAGG
TRAC	GACCAGCTTGACATCACAG	TGTTGCTCTTGAAGTCCATAG
TRBV2	TCGATGATCAATTCTCAGTTG	TTCACTCTGAAGATCCGGTC
TRBV3-1	CAAAATACCTGGTCACACAG	AATCTTCACATCAATTCCCTG
TRBV4-1, 4-2, 4-3	TCGCTTCTCACCTGAATG	CCTGCAGCCAGAAGACTC
TRBV5-1, 5-3, 5-4	GATTCTCAGGKCKCCAGTTC	CTTGGAGCTGGRSGACTC
TRBV5-5, 5-6, 5-7, 5-8	GTACCAACAGGYCCTGGGT	TCTGAGCTGAATGTGAACG
TRBV6-1, 6-2, 6-3, 6-5, 6-6, 6-7, 6-8, 6-9	ACTCAGACCCCAAAATTCC	GTGTRCCCAGGATATGAACC
TRBV6-4	ACTGGCAAAGGAGAAGTCC	TGGTTATAGTGTCTCCAGAGC
TRBV7-1, 7-2, 7-3	TRTGATCCAATTTCAGGTCA	TCYACTCTGAMGWTCCAGCG
TRBV7-4, 7-6, 7-7, 7-8, 7-9	CGSWTCTYTGCAGARAGGC	TGRMGATYCAGCGCACA
TRBV9	GATCACAGCAACTGGACAG	GTACCAACAGAGCCTGGAC
TRBV10-1	CAGAGCCCAAGACACAAG	TGGTATCGACAAGACCTGG
TRBV10-2	ACCTTGATGTGTCACCAGAC	GGAACACCAGTGACTCTGAG
TRBV11-1, 11-2, 11-3	CGATTTTCTGCAGAGACGC	GACTCCACTCTCAAGATCCA
TRBV12-3, 12-4, 12-5	ARGTGACAGARATGGGACAA	CYACTCTGARGATCCAGCC
TRBV13	AGCGATAAAGGAAGCATCC	CATTCTGAACTGAACATGAGC
TRBV14	CCAACAATCGATTCTTAGCTG	ATTCTACTCTGAAGGTGCAGC
TRBV15	AGTGACCCTGAGTTGTTCTC	ATAACTTCCAATCCAGGAGG
TRBV16	GTCTTTGATGAAACAGGTATGC	CTGTAGCCTTGAGATCCAGG
TRBV17	CAGACCCCCAGACACAAG	TGTTCACTGGTACCGACAG
TRBV18	CATAGATGAGTCAGGAATGCC	CGATTTTCTGCTGAATTTCC
TRBV19	AGTTGTGAACAGAATTTGAACC	TTCCTCTCACTGTGACATCG
TRBV20-1	AAGTTTCTCATCAACCATGC	ACTCTGACAGTGACCAGTGC
TRBV23-1	GCGATTCTCATCTCAATGC	GCAATCCTGTCCTCAGAAC
TRBV24-1	CCTACGGTTGATCTATTACTCC	GATGGATACAGTGTCTCTCGA
TRBV25-1	ACTACACCTCATCCACTATTCC	CAGAGAAGGGAGATCTTTCC
TRBV27, 28	TGGTATCGACAAGACCCAG	TTCYCCCTGATYCTGGAGTC
TRBV29-1	TTCTGGTACCGTCAGCAAC	TCTGACTGTGAGCAACATGAG
TRBV30	TCCAGCTGCTCTTCTACTCC	AGAATCTCTCAGCCTCCAGAC
TRBC	TAGAACTGGACTTGACAGCG	TTCTGATGGCTCAAACACAG

Primers for *TRAV* and *TRBV* genes are sense. Primers for *TRAC* and *TRBC* genes are antisense. *TRAV* =T cell receptor Va gene; *TRAC* =T cell receptor Ca gene; *TRBV* = T cell receptor Vb gene; *TRBC* = T cell receptor Cb gene. Supplementary Table 8 is adapted from: Wang GC, Dash P, McCullers JA, Doherty PC, Thomas PG. T cell receptor alphabeta diversity inversely correlates with pathogen-specific antibody levels in human cytomegalovirus infection. Sci Transl Med 4, 128ra142 (2012).
