# Supplementary material for: T cell receptor recognition of hybrid insulin peptides bound to HLA-DQ8
Source: Nat Commun. 2021 Aug 25;12:5110. doi: 10.1038/s41467-021-25404-x (PMC8387461; doi:10.1038/s41467-021-25404-x)
Supplement: Supplementary file 2 — Description of Additional Supplementary Files [file 41467_2021_25404_MOESM2_ESM.pdf]

Description of Additional Supplementary Files

File Name: Supplementary Data 1

Description: **Identified HLA DQ-8 bound linear and spliced peptides. Sheet 1 peptides List of all peptides** identified including mass characteristics and the source protein **Sheet 2 Length Analysis** distribution analysis of peptides based of length **Sheet 3 10-20mer Peptides** list of peptides of 10 to 20 amino acids in length for NetMHCII analysis **Sheet 4 PTM Analysis** identification of peptides with post-translational modifications and the nature of those modifications **Sheet 5 NetMHCII** analysis of peptides using NetMHCII to determine the proportion of peptides binding to HLA-DQ8.
