# Supplementary material for: T cell receptor recognition of hybrid insulin peptides bound to HLA-DQ8
Source: Nat Commun. 2021 Aug 25;12:5110. doi: 10.1038/s41467-021-25404-x (PMC8387461; doi:10.1038/s41467-021-25404-x)
Supplement: Supplementary file 4 — Reporting Summary [file 41467_2021_25404_MOESM4_ESM.pdf]

nature research

Corresponding author(s): Jamie Rossjohn
Hugh H. Reid

Last updated by author(s): Jul 7, 2021

Reporting Summary

Nature Research wishes to improve the reproducibility of the work that we publish. This form provides structure for consistency and transparency in reporting. For further information on Nature Research policies, see Authors & Referees and the Editorial Policy Checklist.

Statistics

For all statistical analyses, confirm that the following items are present in the figure legend, table legend, main text, or Methods section.

n/a	Confirmed	
☐	☒	The exact sample size (*n*) for each experimental group/condition, given as a discrete number and unit of measurement
☐	☒	A statement on whether measurements were taken from distinct samples or whether the same sample was measured repeatedly
☐	☒	The statistical test(s) used AND whether they are one- or two-sided<br>*Only common tests should be described solely by name; describe more complex techniques in the Methods section.*
☒	☐	A description of all covariates tested
☒	☐	A description of any assumptions or corrections, such as tests of normality and adjustment for multiple comparisons
☐	☒	A full description of the statistical parameters including central tendency (e.g. means) or other basic estimates (e.g. regression coefficient) AND variation (e.g. standard deviation) or associated estimates of uncertainty (e.g. confidence intervals)
☐	☒	For null hypothesis testing, the test statistic (e.g. *F*, *t*, *r*) with confidence intervals, effect sizes, degrees of freedom and *P* value noted<br>*Give P values as exact values whenever suitable.*
☒	☐	For Bayesian analysis, information on the choice of priors and Markov chain Monte Carlo settings
☒	☐	For hierarchical and complex designs, identification of the appropriate level for tests and full reporting of outcomes
☒	☐	Estimates of effect sizes (e.g. Cohen's *d*, Pearson's *r*), indicating how they were calculated

Our web collection on statistics for biologists contains articles on many of the points above.

Software and code

Policy information about availability of computer code

Data collection	All Mass Spectrometry data were acquired using Orbitrap Tribrid MS Series Instrument Control Software Version 3.3 (ThermoScientific, San Jose, CA, USA). FACS data: BD FACSDiva-8.0.1. Crystallography data: collection code implemented at the Australian Synchrotron MX1 and MX2 beamlines, Analyst® TF 1.8 (SCIEX).
Data analysis	For Mass Spectrometry: Data were analysed using PEAKS Studio Xplus (Bioinformatics Solutions Inc, Waterloo, Canada) with the following settings: parent mass error tolerance of 10ppm; fragment mass error tolerance of 0.02Da; no enzyme cleavage; variable modifications of oxidation (M). Data were searched against the human proteome (Uniprot, November 2018). PEAKS PTM, was subsequently performed in which unassigned spectra were searched against the human proteome (Uniprot, November 2018) database by including 55 common modifications with a FDR cut-off of 1% applied. The top 20 high confidence de novo sequenced candidates without any linear peptide match were further interrogated with the “Hybrid finder” algorithm (Faridi et al. (2018) A prominent subset of HLA-I peptides are not genomically templated: evidence for cis- and trans-spliced peptide ligands, Science Immunology, 3(28). pii: eaar3947) and the identified cis- and trans-spliced candidate sequences added back to the original database. A “Multi-Run Search with Denovo Only Spectra” was performed by using the combined database. Linear and spliced peptides in this search were extracted at 1% FDR to create the final list of identified peptides; For crystallographic procedures: phenix-1.14-3260, ccp4-7.0, coot-8.5, xds-20190315,;All crystallographic figures were generated using PyMol V2.3.2; For SPR: Prism-9 (Graph pad); For FACS analysis: FlowJo-10.6.0 (Tree Star); For single cell TCR TRAV/TRBV usage analysis: databank search engines IMGT/V-QUEST (http://www.imgt.org/IMGT_vquest/vquest?livret=0&Option=humanTcR). For circular dichroism (CD) spectroscopy: CD Analysis & Plotting Tool (https://capito.uni-jena.de).

For manuscripts utilizing custom algorithms or software that are central to the research but not yet described in published literature, software must be made available to editors/reviewers. We strongly encourage code deposition in a community repository (e.g. GitHub). See the Nature Research guidelines for submitting code & software for further information.

Data

Policy information about availability of data

All manuscripts must include a data availability statement. This statement should provide the following information, where applicable:

- Accession codes, unique identifiers, or web links for publicly available datasets
- A list of figures that have associated raw data
- A description of any restrictions on data availability

Data availability. The mass spectrometry proteomics data generated in this study have been deposited to the ProteomeXchange Consortium via the PRIDE (https://www.ebi.ac.uk/pride/) partner repository with the dataset identifier PXD019466 (http://www.ebi.ac.uk/pride/archive/projects/PXD019466). The structures and structure factors for the complexes of HLA-DQ8-L11C with TCRs A2.13, A1.9 and A3.10 generated in this study have been deposited at the Worldwide Protein Data Bank (wwPDB) under accession codes PDB 6XCP (PDB DOI: https://doi.org/10.2210/pdb6XCP/pdb), 6XCO (PDB DOI: https://doi.org/10.2210/pdb6XCO/pdb) and 6XC9 (PDB DOI: https://doi.org/10.2210/pdb6XC9/pdb), respectively. The T cell stimulation (Supplementary Figure 1) and SPR (Figure 3 and Supplementary Figures 3, 4 and 10) data generated in this study are provided in the Supplementary Information/Source Data file.

Field-specific reporting

Please select the one below that is the best fit for your research. If you are not sure, read the appropriate sections before making your selection.

☒ Life sciences ☐ Behavioural & social sciences ☐ Ecological, evolutionary & environmental sciences

For a reference copy of the document with all sections, see nature.com/documents/nr-reporting-summary-flat.pdf

Life sciences study design

All studies must disclose on these points even when the disclosure is negative.

Sample size	No sample size calculations were undertaken. Sample sizes for SPR and T cell stimulation experiments were chosen based on observed reproducibility of experimental outcomes in preliminary experiments: For SPR $n \geq 2$ independent experiments with $n \geq 2$ technical replicates performed. For T cell stimulation assays $n > 3$ independent experiments with $n = 2$ technical replicates performed for each data point. These sample sizes have been previously used by our laboratory: Petersen et al Nat Struct Mol Biol. 2020 Jan;27(1):49-61. doi: 10.1038/, s41594-019-0353-4; Beringer et al Nat Immunol. 2015 Nov;16(11):1153-61.doi: 10.1038/ni.3271; For tissue samples (PBMC) isolated from T1D patients: No sample size calculations were performed. The number of samples tested depending upon the number of participants with recent onset T1D that could be recruited to the study.
Data exclusions	No data exclusion.
Replication	Each experiment was performed at least twice independently as indicated in the figure legends.
Randomization	Randomisation was not relevant to this study because it was in vitro biochemical based analysis and was not an experimental study that required allocation into groups.
Blinding	Not relevant. This was not a clinical study so no cohort comparison (i.e. different treatment groups) or similar experiment was performed.

Reporting for specific materials, systems and methods

We require information from authors about some types of materials, experimental systems and methods used in many studies. Here, indicate whether each material, system or method listed is relevant to your study. If you are not sure if a list item applies to your research, read the appropriate section before selecting a response.

Materials & experimental systems

n/a	Involved in the study	
☐	☒	Antibodies
☐	☒	Eukaryotic cell lines
☒	☐	Palaeontology
☒	☐	Animals and other organisms
☐	☒	Human research participants
☒	☐	Clinical data

Methods

n/a	Involved in the study	
☒	☐	ChIP-seq
☐	☒	Flow cytometry
☒	☐	MRI-based neuroimaging

Antibodies

Antibodies used	V450 mouse anti-human CD3 (clone UCHT1, cat. no. 561812, BD Biosciences); APC mouse anti-human CD4 (clone RPA-T4, cat. no. 555349, BD Biosciences); BUV395 mouse anti-human CD3 (clone UCHT1, cat. no. 563546, BD Biosciences); APC Mouse Anti-

Human CD69 (Clone FN50, cat no. 555533, BD Biosciences); Alexa Fluor 647 mouse anti-human CD4 (clone OKT4 (IgG2b); Walter and Eliza Hall Institute mAb Facility); anti-HLA-DQ (clone SPV-L3).

Validation

V450 mouse anti-human CD3 (clone UCHT1, cat. no. 561812, BD Biosciences), BUV395 mouse anti-human CD3 (clone UCHT1, cat. no. 563546, BD Biosciences):
Barclay NA, Brown MH, Birkeland ML, et al, ed. The Leukocyte Antigen FactsBook. San Diego, CA: Academic Press; 1997(Biology)Beverley PC, Callard RE. Distinctive functional characteristics of human "T" lymphocytes defined by E rosetting or a monoclonal anti-T cell antibody. Eur J Immunol. 1981; 11(4):329-334. (Biology)Burns GF, Boyd AW, Beverley PC. Two monoclonal anti-human T lymphocyte antibodies have similar biologic effects and recognize the same cell surface antigen. J Immunol. 1982; 129(4):1451-1457. (Clone-specific: Blocking, Functional assay, Immunoprecipitation, Inhibition, Radioimmunoassay)Knapp W, Dörken B, Gilks WR, et al, ed. Leucocyte Typing IV. New York, NY: Oxford University Press; 1989:1-1182. (Clone-specific)Lanier LL, Allison JP, Phillips JH. Correlation of cell surface antigen expression on human thymocytes by multi-color flow cytometric analysis: implications for differentiation. J Immunol. 1986; 137(8):2501-2507. (Biology)McMichael AJ, Beverly PCL, Gilks W, et al, ed. Leukocyte Typing III: White Cell Differentiation Antigens. New York: Oxford University Press; 1987:1-1050. (Clone-specific)Schlossman SF, Boumsell L, Gilks W, et al, ed. Leucocyte Typing V. New York: Oxford University Press; 1995(Biology)Schlossman SF, Boumsell L, Gilks W, et al, ed. Leukocyte Typing V: White Cell Differentiation Antigens. Oxford: Oxford University Press; 1995(Clone-specific)Van Wauwe JP, Goossens JG, Beverley PC. Human T lymphocyte activation by monoclonal antibodies; OKT3, but not UCHT1, triggers mitogenesis via an interleukin 2-dependent mechanism. J Immunol. 1984; 133(1):129-132. (Clone-specific: Flow cytometry, Functional assay, Stimulation);

APC mouse anti-human CD4 (clone RPA-T4, cat. no. 555349, BD Biosciences); APC Mouse Anti-Human CD69 (Clone FN50, cat no. 555533, BD Biosciences):
Knapp W, Dorken B, Rieber EP, et al, ed. Leucocyte Typing IV. New York: Oxford University Press; 1989:1-1208. (Clone-specific)Schlossman SF, Boumsell L, Gilks W, et al, ed. Leukocyte Typing V: White Cell Differentiation Antigens. New York: Oxford University Press; 1995. (Clone-specific).

Alexa Fluor 647 mouse anti-human CD4 (clone OKT4 (IgG2b); WEHI Antibody Facility; https://www.wehi.edu.au/research/research-technologies/antibody-technologies):
Kung P, Goldstein G, Reinherz EL, Schlossman SF. Monoclonal antibodies defining distinctive human T cell surfaceantigens. Science. 1979; 206(4416):347-349. (Immunogen: Cytotoxicity, Flow cytometry, Radioimmunoassay).Horibe K, Knowles RW, Naito K, Morishima Y, Dupont B. Analysis of T lymphocyte antibody specificities: Comparison ofserology with immunoprecipitation patterns. In: Bernard A. A. Bernard .. et al., ed. Leucocyte typing : human leucocytedifferentiation antigens detected by monoclonal antibodies. Berlin New York: Springer-Verlag; 1984:212-224.Moebius U. Cluster report: CD4. In: Knapp W. W. Knapp .. et al., ed. Leucocyte typing IV : white cell differentiation antigens.Oxford New York: Oxford University Press; 1989:314-330.Miedema F, Terpstra FG, Melief CJM. T Cell-dependent immunoglobulin synthesis in the human system. Studies with T cell-specific monoclonal antibodies. In: Reinherz EL. Ellis L. Reinherz .. et al., ed. Leukocyte typing II. New York: Springer-Verlag;1986:213-222.Reinherz EL, Kung PC, Goldstein G, Levey RH, Schlossman SF. Discrete stages of human intrathymic differentiation: analysisof normal thymocytes and leukemic lymphoblasts of T-cell lineage. Proc Natl Acad Sci U S A. 1980; 77(3):1588-1592.(Clone-specific: Cell separation, Cytotoxicity, Flow cytometry).Reinherz EL, Kung PC, Goldstein G, Schlossman SF. Further characterization of the human inducer T cell subset defined bymonoclonal antibody. J Immunol. 1979; 123(6):2894-2896. (Clone-specific: Flow cytometry, Fluorescence activated cellsorting).Reinherz EL, Kung PC, Goldstein G, Schlossman SF. Separation of functional subsets of human T cells by a monoclonalantibody. Proc Natl Acad Sci U S A. 1979; 76(8):4061-4065. (Immunogen: Cell separation, Flow cytometry, Fluorescenceactivated cell sorting).Hoffman RA, Kung PC, Hansen WP, Goldstein G.. Simple and rapid measurement of human T lymphocytes and theirsubclasses in peripheral blood.. Proc Natl Acad Sci U S A. 1980; 77(8):4914-4917. (Clone-specific: Flow cytometry).
750972 Rev. 3Page 3 of 3Courtney AH, Lo WL, Weiss A. TCR Signaling: Mechanisms of Initiation and Propagation. Trends Biochem Sci. 2018;43(2):108-123. (Biology).

Anti-HLA-DQ (clone SPV-L3):
PubMed=6332061; DOI=10.1089/hyb.1983.2.423
Spits H., Keizer G., Borst J., Terhorst C., Hekman A., de Vries J.E.
Characterization of monoclonal antibodies against cell surface molecules associated with cytotoxic activity of natural and activated killer cells and cloned CTL lines.
Hybridoma 2:423-437(1983)

PubMed=6609821; DOI=10.1002/eji.1830140404
Spits H., Borst J., Giphart M., Coligan J., Terhorst C., de Vries J.E.
HLA-DC antigens can serve as recognition elements for human cytotoxic T lymphocytes.
Eur. J. Immunol. 14:299-304(1984).

A.A. te Velde, J.P.G. Klomp, B.A. Yard, J.E. de Vries, C.G. Figdor. Modulation of phenotypic and functional properties of Human peripheral blood monocytes by IL-4. J. of Immunol. 1988, 140: 1548-1554.

Eukaryotic cell lines

Policy information about cell lines

Cell line source(s)

HEK293T cells were from ATCC. BLCL lines were sourced from the The International Histocompatibility Working Group (IHWG) Cell and DNA Bank, SKW3 line was sourced from German Collection of Microorganisms and Cell Cultures (DSMZ). High Five Cells (BTI-TN-5B1-4) were from Thermo Fisher Scientific.

Authentication

HLA-DQ expression was confirmed on 9033 BLCL cells by staining with anti-DQ monoclonal antibodies and FACS analysis. CD4 expression and absence of CD3 expression was confirmed on SKW3 parental cells by staining with anti-CD4 and anti-CD3

	monoclonal antibodies, respectively, and FACS analysis. T cell receptor (TCR) transduction of SKW3 cells was confirmed by IRES driven expression of GFP- and RFP-reporter proteins, (TCR a-chain and TCR b-chain, respectively) and were antibody stained to confirm CD3 (indicating TCR surface expression). HEK293T cells were from ATCC and High Five Cells (BTI-TN-5B1-4) from Thermo Fisher Scientific were not authenticated.
Mycoplasma contamination	Absence of mycoplasma contamination in cell lines was confirmed via PCR.
Commonly misidentified lines (See ICLAC register)	No commonly misidentified cell lines were used in the study.

Human research participants

Policy information about studies involving human research participants

Population characteristics	Participants were selected based solely on a recent diagnosis of type 1 diabetes. Peripheral blood samples were collected from participants within 100 days of diagnosis with T1D. Age range from 4 to 18 years of age.
Recruitment	Participants were recruited from type 1 diabetes clinic at the Royal Children's hospital Melbourne. All participants had recent onset (within 6 weeks of diagnosis) type 1 diabetes and were aged between 5 and 18 years of age. Only participants who agreed to participate were included in the study, otherwise there we no self-selection biases.
Ethics oversight	Ethical approval was provided by the Southern Health Research Ethics Committee (HREC Reference number: 12185B) and the Royal Children's Hospital Research Ethics committee (SSA No.SSA/36346, RCH HREC No. 36346).

Note that full information on the approval of the study protocol must also be provided in the manuscript.

Flow Cytometry

Plots

Confirm that:

☒ The axis labels state the marker and fluorochrome used (e.g. CD4-FITC).

☒ The axis scales are clearly visible. Include numbers along axes only for bottom left plot of group (a 'group' is an analysis of identical markers).

☒ All plots are contour plots with outliers or pseudocolor plots.

☒ A numerical value for number of cells or percentage (with statistics) is provided.

Methodology

Sample preparation	Cells were harvested by centrifugation and resuspended/washed with Phosphate buffered saline (PBS). 2% fetal bovine serum was used for antibody and HLA-DQ8 tetramer staining. For HLA-DQ8 tetramer staining of TCR transduced SKW3 cells, cells were fixed with 2% fomaldehyde in PBS prior to analysis.
Instrument	BD LSRFortessa™ X-20, BD FACS ARIA Fusion
Software	FlowJo v10
Cell population abundance	For single-cell sorting samples a 'post-sort' flow cytometry analysis was not possible.
Gating strategy	HLA-DQ8-HIP restricted TCR (GFP/RFP) transduced SKW3 cells were gated as follows: Lymphocytes (FCS-A/SSC-H); single cells (FSC-A/FSC-H); live cells (AQUA stain, BV525-A low); TCR:GFP expression (B530-A high), RFP expression (YG586 high). HLA-DQ8-HIP restricted cells (APC-HLA-DQ8-HIP tetramer (R670) and V450-CD3 (V450) vs PE-HLA-DQ8-gliadin tetramers and V450-CD3 (V450) -ve control). Single cell HLA-DQ8-HIP tetramer sorted cells were gated as follows: : Lymphocytes (SSC/FSC); single cells (FSC/FSC-A); live cells (Propidium Iodide, FSC),CD4+ cells (AF-647-CD4), HLA-DQ8-HIP restricted T cells (PE-HLA-DQ8-HIP tetramers/CFSE low vs PE-HLA-DQ8-gliadin tetramers/CFSE low -ve control)

☒ Tick this box to confirm that a figure exemplifying the gating strategy is provided in the Supplementary Information.
